# Multi-centennial mass balance of perennial ice deposits in Alpine caves mirrors the evolution of glaciers during the Late Holocene

**DOI:** 10.1038/s41598-022-15516-9

**Published:** 2022-07-05

**Authors:** Tanguy M. F. Racine, Paula J. Reimer, Christoph Spötl

**Affiliations:** 1 Institute of Geology, University of Innsbruck, Innrain 52f, 6020 Innsbruck, Austria; 2 Queens University Belfast, Belfast, BT7 1NN UK

**Keywords:** Cryospheric science, Palaeoclimate

## Abstract

Mid-latitude alpine caves preserve a record of past solid precipitation during winter, locally spanning several centuries to millennia. Dating organic macro-remains trapped in ice layers allows the determination of timing and duration of past periods of positive and negative ice mass balance. We present here the largest comparative study of ice cave sites yet published, using Bayesian age-modelling on a database comprising 107 radiocarbon dates, spread over eight caves in the Austrian Alps. We show that periods of positive mass balance coincide with past glacier advances. We find organic and macro-remain rich layers dated to the Medieval Climate Anomaly (between 850 and 1200 CE) marking widespread ice retreat. We demonstrate positive ice mass balance at all studied sites for the Little Ice Age, coinciding with the largest glacier advances in the Holocene between 1400 and 1850 CE. At the sites with records spanning over 2000 years, positive mass balance is also observed during the periods from 300 BCE to 100 CE and 600–800 CE. These subterranean ice deposits show widespread evidence of accelerated negative mass balances in recent years and their record is under imminent threat of disappearing.

## Introduction

Glaciers worldwide have experienced overwhelmingly negative mass balance in the early 21st Century, with thinning rates doubling over the period 2000–2020^1^. In the European Alps, modelling studies consistently point towards the near-total loss of glaciers by the end of the 21st Century^2–4^. Ice deposited and preserved underground in alpine caves is likewise experiencing a widespread retreat in many parts of the European Alps and mountain regions elsewhere^5–7^. Such rock-hosted caves where perennial ice or snow occur are known as ice caves^8^.

Although several thousands of ice caves are known and documented worldwide^8^, no more than a few dozen have been investigated in detail^9–13^. Ice deposits in underground caverns are generated by the firnification of snow entering the cave during the cold season or the freezing of water, or both, and preserved thanks to specific ventilation patterns which cool the cave environment^14,15^. A growing body of evidence, ranging from monitoring ice-level fluctuations^16^, radiocarbon-dating of organic matter included in ice^10,17^, trace element^11^ and stable isotope analyses^18,19^ demonstrates the clear paleoclimatic potential of underground ice archives, as well as their high vulnerability in a changing climate^5^.

Caves with a descending morphology, open pits, also known as sag-type, collect winter snow, whose accumulation and subsequent firnification builds an underground ice body^15^. The present study focuses on underground ice deposits in such sag-type caves because they offer the most direct link to atmospheric processes and act as natural snow collectors during the cold season. Leaves, twigs, seeds and cones, or fragments thereof, sourced from the surrounding vegetation are transported into the cave where they may eventually become embedded in the accumulating snow. This process leads to stratified ice bodies typically a few metres to a few tens of metres in thickness, containing inclusions of various origin, organised in layers^12,20^. Ice accumulated in this way can be lost through melting or sublimation occurring at the different ice-water, ice-air or ice-rock interfaces. Ice mass balance in sag-type caves is thus a complex function of precipitation type (snow or rain), its temporal distribution and amount, as well as rock and air temperature profiles. Like surface glaciers, this underground ice represents the time integration of a variable annual mass balance^21^.

Estimates of past mean annual mass balance (MAMB) can be derived from age-depth relationships or chronostratigraphies of underground ice deposits. Placing mass balance changes in a chronological context requires the age of the ice to be determined^22^, a task previously achieved using annual layer counting wherever feasible^23^ or radiogenic ^210^Pb age determinations for ice deposits no older than about 150 years^17,24^. Most studies, however, rely on radiocarbon age determination of wood inclusions for dating underground ice deposits^22^. The oldest wood macro remains found in ice caves of the European Alps are dated from 3366 to 3030 BCE (95.6%)^25,26^, and the oldest currently known sample from an alpine ice cave worldwide, dated from 4256 to 4043 BCE (95.6%), was found in a Pyrenean cave^12^.

Past decades have seen a slowly rising number of studies of chronostratigraphies at cave sites in the Alps^9,13^ and in other mountain chains of Europe^10,12,16,27^ and North America^11,28^. To date, most published ice cave records focus on single cave ice deposits. For instance, a combination of radiocarbon-dating and dendrochronology was used to reconstruct past periods of positive ice mass balance at St. Livres ice cave^9^, while the age of the ice in the nearby Monlési Ice Cave was constrained by ^210^Pb, ^14^C and anthropogenic inclusions^17^, both ice caves being located in the Jura Mountains of northern Switzerland. At Hundsalm Ice Cave, western Austria, periods of positive and negative mass balance were bracketed using a 19-sample radiocarbon dataset from wood inclusions^13^. Similarly, the timing and duration of ice deposition in a sag-type cave of the Julian Alps were constrained with the aid of an 18-sample dataset^29^. At Dobšínská cave in Slovakia, successive campaigns sampling the remains of bats embedded in the ice body helped constrain the age of the ice deposit to 2600 years^10,30,31^. In the central Pyrenees, the record of 22 radiocarbon samples from A294 cave was shown to span several millennia^12^, and similarly, the exceptional record of Scărișoara ice cave, Romania, has been the subject of several publications, linking stable isotopes of ice to past winter climate variability during the Holocene^20,32^. The ice in Strickler Cavern (Idaho, USA) was shown to span the past 2000 years based on 26 radiocarbon samples^11^. There is therefore an increasing need to broaden the database and to compare and integrate records from different sites in order to better understand the response of this part of the cryosphere to climate forcing.

Karst regions of the European Alps are particularly conducive to the development of underground ice, in no small part due to the significant amount of snow in winter and the large number of vertical pits developed in carbonate rocks outcropping at elevations over 1500 m a. s. l.^26^. Many sites also benefit from having been explored and mapped several decades ago, and thus provide a benchmark for more recent ice- and snow-level variations. As such, the Austrian Alps, constituting the eastern part of the European Alps, are ideally suited for such a regional study. Using a total of 107 radiocarbon-dated woody macroremains, this study reports the first detailed chronostratigraphy at eight caves, providing a simultaneous comparison of the ice mass balance dynamics in those caves.

Owing to the large limestone plateaus in the Northern Calcareous Alps, where most of the caves are situated at elevations ranging 1500–2100 m a.s.l., more than 1200 ice-bearing caves are recorded in the Austrian cave cadastre^26^. Within this database, sag-type caves containing a clearly visible and accessible stratigraphy, where the presence of wood debris was attested from previous speleological documentation, made the best targets.

**Figure 1:**
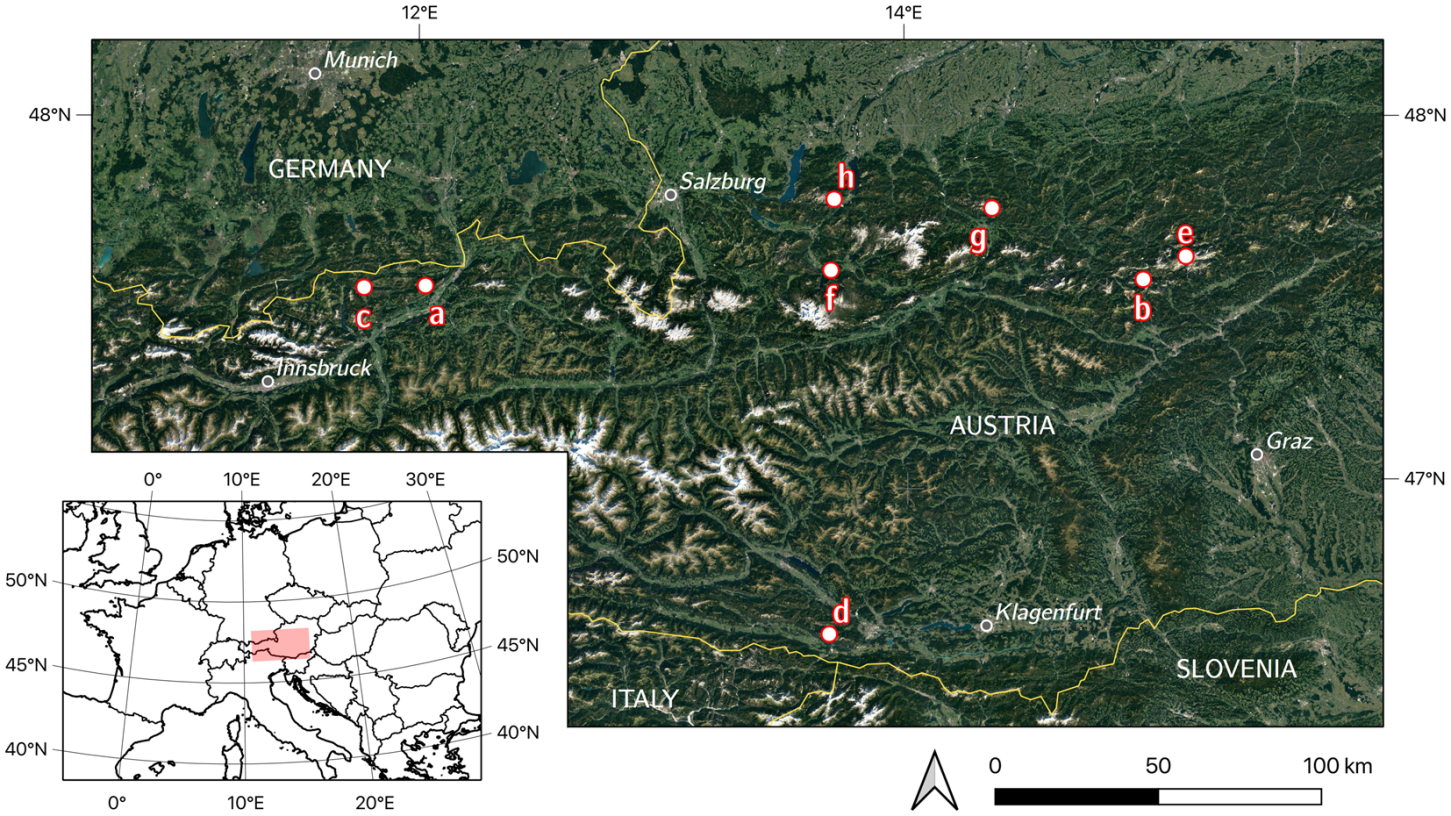
Studied caves in the Eastern Alps of Austria. (a) Hundsalm Eis- und Tropfsteinhöhle, (b) Bärenloch Eishöhle, (c) Guffert Eisschacht, (d) Großer Naturschacht, (e) Tremml-Schacht-413, (f) Eisgruben Eishöhle, (g) Kraterschacht, (h) Hochschneid Eishöhle. Google Satellite 2022.

**Figure 2:**
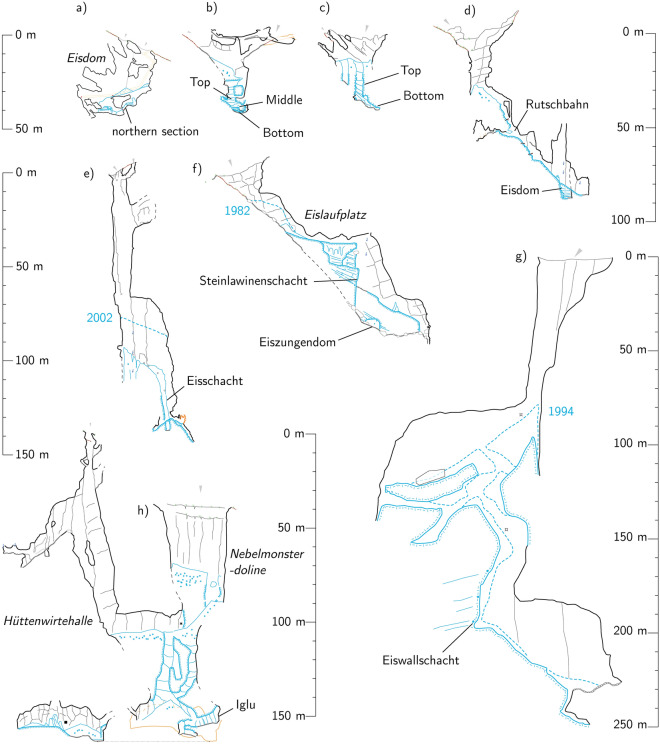
Extended profiles of the studied sites. (**a**) Hundsalm Eis- und Tropfsteinhöhle, (**b**) Bärenloch Eishöhle (**c**) Guffert Eisschacht, (**d**) Großer Naturschacht, redrawn after Jenatschke et al.^61^, (**e**) Tremml-Schacht-413, (**f**) Eisgruben Eishöhle, (**g**) Kraterschacht, redrawn after Weißmair et al.^35^ (**h**) Hochschneid Eishöhle, redrawn after Wielander et al.^62^. Steepled blue lines denote snow or firn levels recorded previously.

The caves selected for this study (Fig. 1) are found between 1518 m a.s.l. (Bärenloch Eishöhle) and 2100 m a.s.l. (Großer Naturschacht). All are located below their respective local tree lines, variously defined by dwarf pine (*Pinus mugo*), spruce (*Picea abies*) and larch (*Larix decidua*). All caves are of the sag-type geometry (Fig. 2), i.e., they have one large entrance with steeply descending passage where winter snow is deposited. Snow and scattered woody macro-remains entering the caves by such openings build up a layered deposit containing alternating clean ice units and layers with more concentrated organic matter. Owing to the proximity of these underground ice bodies to the surface, and the nature of the karstified rock, snow deposited within the cave is wetted by dripwater or rain, a process which accelerates the transformation of firn into ice, by the freezing of water in snow voids^9,15^. Fragments of the cave host rock, cryoclasts, are also commonly incorporated in such underground ice deposits. The regular alternation of positive and negative mass balance periods leads to the build-up of horizons rich in cryoclasts and wood remains. The resulting geometries are analogous to angular unconformities, where the ice layers in units above and below the wood-rich boundary are not parallel (e.g., in Eisgruben, Figs. A.1C & A.2B). This geometry indicates centennial to multi-centennial periods of mass loss. Paraconformities refer to parallel ice layering (Fig. A.1B) concealing shorter (decadal to multi-decadal) mass loss episodes which did not result in a significantly altered ice surface.

The aim of this study is to retrieve and date many woody material inclusions found in 2D exposures of ice. Thus constraining the temporal span of stratigraphic units called sections, delimited by accessibility or large unconformities, we provide evidence for past mean positive mass balance during the time covered by that particular section. Building a history of ice mass balance for each cave, we compare the data from eight different caves with the length changes of their surface glacier counterparts.

## Results

In total, 107 radiocarbon samples, spread over eight caves and their ice exposures were analysed and placed in a stratigraphic context (Extended Data A). For each cave, an age-depth model was established, thereby providing constraints on the mass balance dynamics at these sites (Extended Data B). At the sites of Guffert, Eisgruben and Bärenloch, the compiled age lists and respective age-depth models reach back before the Common Era and provide strong evidence for positive MAMB prior to the Little Ice Age (LIA, 1260 CE to 1860 CE^33^). At Hundsalm, there is also evidence for positive MAMB^13^ These four caves were continuously glaciated during the last two millennia. Within the other caves, the radiocarbon data give the earliest evidence of continuous glaciation either in the early LIA The comparison of modern observations with previous exploration reports, topometric surveys and photographs reveals a negative MAMB for the last three decades at least in the majority of the study sites.

### Hundsalm Eisund Tropfsteinhöhle

We re-sampled the *northern section* of this cave adding five samples to the five samples of a previous study^13^. One sample yielded modern radiocarbon age and another sample was identified as an outlier, leaving eight samples to model this section (Extended Data B.1). In contrast to the section previously sampled near *Tiefster Punkt*, which lacks a stratigraphic progression^13^, the eight samples from the *northern section* are in stratigraphic order (Fig. B.1). Three samples bracket a period of positive MAMB spanning 600 CE to 800 CE (Table S1). At that time, the age model suggests a MAMB of about 1 cm water equivalent per year (w.e. yr^−1^). These steeply dipping strata are separated from the subsequent discordant unit by a macro-remain-rich unconformity and the next five samples defining a section of positive MAMB spanning 1300 CE to 1700 CE (Table S1), ending at a prominent layer rich in wood remains. The MAMB for this interval is estimated at 1 cm w.e. yr^−1^. The age of the ice located closer to the entrance at a higher stratigraphic position could not be constrained using radiocarbon dating due to the lack of organic matter, but the discovery of the show cave in 1921 CE provides a maximum age for the top of this unit. Little is known about ice development between the discovery of the cave and its opening as a show cave in 1967 CE^13^, but an upper boundary of 1930 ± 10 CE was given in the age model (Extended Data B.1). In light of this constraint, the uppermost part of the *northern section* stratigraphy suggests a MAMB of about 4 cm w.e. yr^−1^. The ice body in this cave is currently experiencing a strongly negative MAMB, as evidenced by the continuous drop in the ice surface of *Eisdom* in the past decades.

### Bärenloch Eishöhle

**Figure 3:**
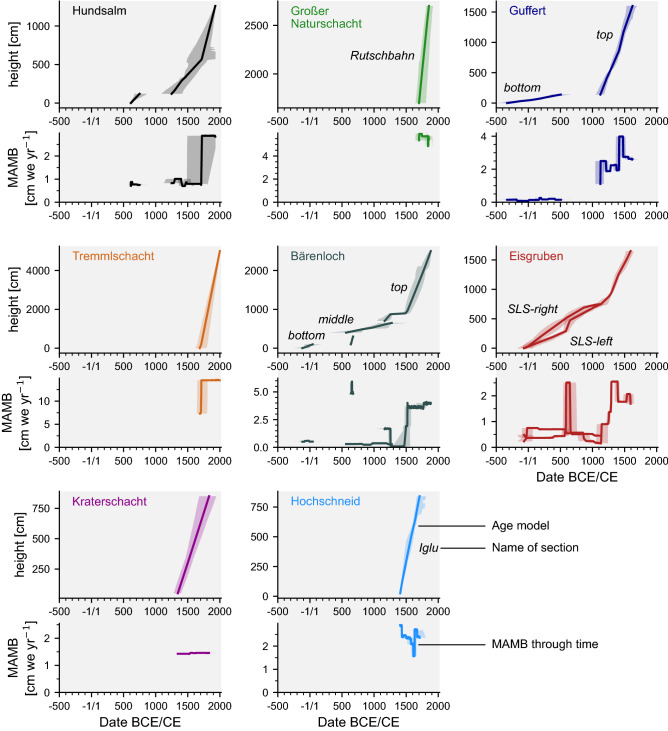
Summary of age-depth modelling and derived MAMB during the last 2.5 millennia all selected caves. Shaded areas denote the 95.6% confidence intervals of the respective age models. At Eisgruben, two sections covering the early part of the record, until Boundary III, were sampled, and thus, two age models and MAMB records were calculated for the relevant time period.

Three separate sections could be accessed and sampled in this 40 m-deep cave (Extended Data A.2, B.2). In the very deepest alcove, 3 m of layered ice are constrained by eight radiocarbon samples (BL-C1 to C8, Fig. B.2). BL-C5 was excluded from the OxCal section model, and the remaining seven samples were modelled as the *bottom section* (Extended Data B.2.3). Samples BL-C1 to C3 span 200 CE to 100 CE (Table S1) with a MAMB of 0.5 cm w.e. yr^−1^ (Fig. 3). The next segment of the model contains four samples defining a closely bracketed section from 600 CE to 700 CE where the MAMB rises more than tenfold to 4.9 cm w.e. yr^−1^ (Fig. 3). Ice deposition continues beyond the last date (BL-C8), but the cave geometry precluded further sampling along the ice section.

In the *middle section*, eight samples were radiocarbon-dated (Fig. B.2), with four younger, out-of-sequence samples identified using the OxCal model (BL-C13,BL-C14, BL-C18, BL-C19). The age model incorporating the remaining four samples (BL-C15, BL-C16, BL-C17 and BL-C21) suggests that this ice section dates from 900 CE to 1300 CE (Table S1). The MAMB averaged 0.3 cm w.e. yr^−1^ (Fig. 3) during this period.

The *top section* consists of seven radiocarbon samples (Fig. B.2, Table S1) and one surface constraint thanks to one late 19th Century observation^34^ (Extended Data B.2). One sample (BL-C24) was rejected from the section (Extended Data B.2.1). The remaining six samples define two main periods of positive mass balance. A large break in the slope of the age model was detected at 1300 CE to 1400 CE.

### Guffert Eisschacht

The geometry of this cave (an ice pit followed by short descending passage, Extended Data A.3) warranted the construction of a separate model for each of the sections (Fig. 2). The *bottom section* corresponds to the innermost, small ice alcove at the bottom of the cave, spanning about 140 cm. A total of seven samples were dated from this short section and all were used to build the age model of the *bottom section* (Fig. B.3, Extended Data B.3.2). The *top section* on the other hand corresponds to the Eisschacht, spanning approximately 15 m, from the bottom up (Fig. 2). A total of 13 samples were dated in this section (Extended Data B.3.1). The outlier analysis revealed clear outliers (GE-C07, GE-C12 and GE-C13), which were excluded (Extended Data B.3.1). The remaining 5 m at the top of the Eisschacht exposure are influenced by the annual snow level variations and show little evidence of stratified ice.

The first of two periods of positive MAMB at Guffert, bracketed by the *bottom section*, started at around 400 BCE, spanned about 800 years (Table S1). Currently, the *bottom section* comprises just over 1 m of ice at the very bottom of the ice pit. The MAMB range between 0.1 and 0.2 cm w.e. yr^−1^ (Fig. 3). The minimum is reached between the 2nd and the 1st Centuries BC, while the maximum occurs at the transition between 2nd and the 3rd Centuries CE. By contrast, the second period, defined by the *top section*, is constrained by the samples taken in the ice pit itself, spans nearly 600 years, from 1200 to 1800 CE. Between about 1100 CE and 1400 CE, the MAMB range from 1 to 2.3 cm w.e. yr^−1^ (Fig. 3). At 1450 CE, the MAMB increase at a maximum of 4 cm w.e. yr^−1^, before dropping again to 2.2 cm w.e. yr^−1^ by 1500 CE. The transition from the *bottom* to the *top section* is therefore characterised by a tenfold increase in MAMB.

### Großer Naturschacht

A total of 15 samples were radiocarbon-dated from this 90 m-deep cave, including nine from the *Rutschbahn section*, and six from the *Eisdom section* (Fig. 2). Samples K5, K7, GNS-C1, GNS-C2, GNS-C3 and GNS-C5 yielded modern radiocarbon values and were excluded from this analysis, leaving seven and two samples in the *Rutschbahn* and *Eisdom sections*, respectively. GNS-C7 and GNS-C9 were excluded as possible outliers (Extended Data B.4), due to their relatively young age with respect to the stratigraphy. The *Rutschbahn section* is comprised of five samples (Fig. B.4).

The ice at the level of GNS-C8 was deposited around the end of the 17th Century CE (Table S1), with some of the underlying ice likely pre-dating it. However, since the very base of the ice is buried under a modern snow cone, no deeper sample could be obtained (Extended Data A.4). Similarly, the age of the ice near sample GNS-C12 is modelled at the 19th Century, and so, the overlying ice and snow must also post-date this. Within the *Rutschbahn section* of about 10 m, deposited over 200 years, the MAMB averages 5.4 cm w.e. yr^−1^ (Fig. 3).

### Tremml-Schacht-413

The age model of this 150 m-deep shaft, hereafter Tremml, consists of two radiocarbon samples (TREM-1 and TREM-2, Table S1) and a surface constraint to be no older than 2002 CE (Extended Data A.5). Since the two samples are located near the base of the deposit, the resulting age model is nearly a linear interpolation between their radiocarbon calibration and the surface constraint (Extended Data B.5). The modelled sequence spans a maximum of 1640 CE to 2000 CE (Table S1), with the highest MAMB of 13.6 cm w.e. yr^−1^ (Fig. 3), compared to the other ice-cave records.

### Eisgruben Eishöhle

The *Eiszungendom section* of this 95 m-deep cave documents positive MAMB between 3700 and 3400 BCE (Fig. B.6a). The upper part of this section extends to about 800 CE (Fig. B.6a), showing some overlap with the *Steinlawinenschacht section* above (Fig. 2). One radiocarbon sample from this sequence was identified as outlier (Table S1 & Fig. B.6a), due to its young age. According to the age model, the MAMB between 3700 and 0 BCE averages below 0.1 cm w.e. yr^−1^.

The 22 m-high *Steinlawinenschacht section* (Fig. 2) yielded 16 radiocarbon samples, of which 13 were used to build the age model. The OxCal model definition separates the model into two parallel sections (SLS-left and SLS-right, Extended Data B.6.2). Wherever the height of common major unconformities could be ascertained, they are incorporated into the model as shared boundaries (I, II and III, Fig. B.6b). The outlier analysis highlighted samples EE-C6, EE-C10 and EE-C18 as potential outliers and they were excluded from the age models (Extended Data B.6.2). The age-depth model of the *Steinlawinenschacht section* documents positive MAMB over the last ≈ 2000 years (Fig. B.6b). The bottom 4–5 m of the section, characterized by numerous and large wood fragments organised in prominent layers, correspond to a period of net, discontinuous ice accumulation spanning the first five Centuries CE (Table S1). The annual mass balance for this period ranges from 0.4 to 0.7 cm w.e. yr^−1^.

The period spanning 600 CE to 700 CE is marked by a seven-fold increase in MAMB recorded on the *Steinlawinenschacht section*, peaking at 2.4 cm w.e. yr^−1^. This peak is only seen in the left-hand section of the exposure due to the angular nature of unconformity II, which has removed part of the record in the SLS-right section. Specifically, the angular unconformity removed a couple metres of ice (bracketed by samples EE-C17 and EE-C19 in SLS-left), yielding a much low mean mass balance for that period on the right.

**Figure 4:**
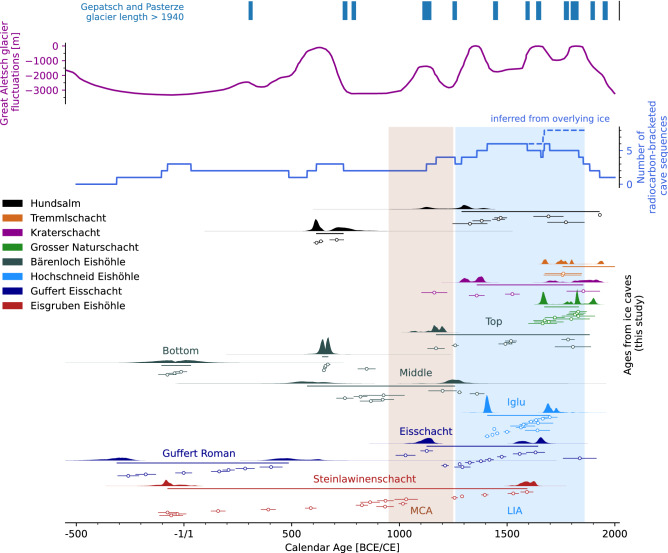
Graphical summary of radiocarbon dates of eight alpine ice caves, plotted alongside past glacier length changes of (**a**) the Lower Grindelwald Glacier^55^, (**b**) the Great Aletsch glacier^43^, (**c**) a number of replicated positive MAMB phases; a count of one was attributed whenever two dated sections from a single cave overlapped, e.g., Eisgruben from 0 to 500 CE, (**d**) summary of the radiocarbon dates; open circles denote median modelled ages, single continuous lines represent the duration of the sections, while areas filled with a solid colour denote the posterior probability density of the starting and ending boundaries. Note that from 1700 to 1850 CE, the number of radiocarbon-bracketed sequences ranges between five and six. This is because the dated sections of Eisgruben, Hochschneid and Guffert end when those of Großer Naturschacht and Tremml-Schacht-413 start. However, given that a large volume of ice was deposited on top of the radiocarbon-dated sequences, we postulate that Hochschneid, Guffert and Eisgruben also experienced positive mass balance for some decades after their youngest radiocarbon samples, bringing the actual total number of cave sequences with positive MAMB to a full eight for the period 1750 CE to 1850 CE.

The three large unconformities identified in the stratigraphy (Extended Data A.6) are evidence of sustained ice retreat bracketing another period of net but clearly discontinuous ice growth spanning the 8th–11th Centuries CE. The deposition of the top half of the stratigraphy, characterised by clear, layered ice with much fewer organic remains started at the turn of the 14th Century CE and continued apparently uninterrupted for at least four centuries (Table S1). The topmost sample (EE-C16) suggests that ice deposition continued at least until the beginning of the 18th Century CE (Fig. 4). The topmost ≈ 5 m of ice stratigraphy were deposited subsequently but owing to the lack of organic inclusions, this part of the ice body could not be precisely constrained. Overall, the MAMB derived from the age model increases three-fold from 1400 CE onwards (dated by the EE-C10 horizon, Fig. B.6b), rising from an average of 0.5 cm w.e. yr^−1^ to about 1.9 cm w.e. yr^−1^ (Fig. 3).

### Kraterschacht

This 250 m-deep cave yielded an age model consisting of three samples and spanning ca. 1400 CE to 1850 CE (Table S1). The age model includes a previously dated wood remain^35^. One sample yielded a modern radiocarbon value (Extended Data B.7) and sample P3 was found to lie outside of stratigraphic order and was thus excluded. The MAMB at Kraterschacht derived from age modelling varies between 1.3 and 1.4 cm w.e. yr^−1^ (Fig. 3).

### Hochschneid Eishöhle

This 163 m-deep cave hosts a well-dated section lacking unconformities and spanning at least 1390 CE to 1770 CE (Fig. 2, Extended Data A.8, Table S1). A total of 12 samples were selected to build the age-depth model (Extended Data B.8). The repeated pattern of thick firn layers poor in macro-remains intercalated with thinner layers rich in such remains (Extended Data A.8) points towards an overall positive MAMB during this period, with sub-centennial episodes of ice loss. On average, a MAMB of 2.3 cm w.e. yr^−1^ was calculated for the section dated from 1390 to 1770 CE (Fig. 3). Above the *Iglu section* the layers thickness increases, and the organic-rich horizons therein are less pervasive and laterally discontinuous. The layering is obscured or lost in the vicinity of the snow-cone of Hüttenwirtehalle (Extended Data A.8). Stratigraphic superposition dictates that this upper part of the ice postdates the *Iglu section* and was therefore deposited between the late 18th Century CE and present.

### Timing and duration of cave glaciation

The start and end point of each ice section in the eight studied caves are displayed in Fig. 4 as a posterior probability density function. The starting boundary of an OxCal P_Sequence describes by no means the first occurrence of ice in that cave, but rather the oldest ice still preserved. This is because the mass turnover rate of a cave puts a limit on the oldest ice that can be preserved and dated^17^. Cave glaciation likely begun before the oldest age. The starting boundary, however, is a minimum age for the beginning of the current state of cave glaciation.

The youngest radiocarbon-dated samples from a section yield an end boundary which cannot be interpreted as marking the cessation of ice deposition, on the contrary. At all sites, the sampling of macro-remains stops when the stratified section transitions upwards into more massive ice and eventually firn and snow. Stratigraphic superposition indicates strongly that ice deposition continued after that last boundary, but with very poor age control and hence, unknown MAMB. The deposition of ice in each cave certainly continued after 1600 CE at Eisgruben, Bärenloch, Guffert and Hochschneid. At Hochschneid, the majority of the ice volume even post-dates the well-dated section found at the bottom. At Bärenloch, the end of the dated, well-layered section is found near the bottom of the cave, again suggesting that more than half of the ice visible in the cave today post-dates this section. At Eisgruben, the undated upper section of the stratigraphy represents nearly 5 m of ice, which post-dates the last sample (EE-C16, at about 1600 CE, Table S1). At Guffert, ice post-dating 1700 CE was also deposited after the youngest radiocarbon-dated sample. The continued ice deposition after the last dated samples is thus a common feature of all studied caves, coinciding with the main LIA phase (1570 CE to 1860 CE)^33^ and continuing thereafter.

The groups of radiocarbon dates underpinning each section thus bracket periods when ice was deposited with variable but on the whole positive MAMB (Fig. 3). Between 1 CE and 500 CE, positive ice mass balance is attested at three sites. The period spanning 1450 CE to 1550 CE saw the coeval deposition of ice in four different caves. A noteworthy common feature of the ice cave records is that from 1550 CE onwards, and for about 300–350 years, there is strong evidence of continued positive MAMB at every studied site. This period is either bracketed by radiocarbon-dated wood inclusions where available (e.g., Naturschacht, Bärenloch, Hundsalm, Tremml), or postulated due to the presence of overlying, organic-poor ice which was deposited after the last radiocarbon-dated sample (e.g., Hochschneid, Eisgruben, Kraterschacht).

### Mean Annual Mass Balance through time

Despite the contrasting geometry of each site, the analysis the eight cave ice records yields a coherent story of mass balance dynamics during the past millennia. It is noteworthy that the elevation difference between the different caves entrances has no obvious impact on the timing or duration of glaciation. Positive MAMB during the period 300 BCE to 500 CE is attested at three of the caves, Eisgruben, Bärenloch and Guffert. At each of those sites, the ice record tied to that period is characterised by a condensed, wood-rich section, which yields MAMB ranging between 0.1 and 0.7 cm w.e. yr^−1^ (Fig. 3).

This coherence continues with Eisgruben and Bärenloch whose MAMB peaks between 600 and 800 CE. At Bärenloch, the transition between the pre-Common Era samples and the 600 CE to 800 CE peak is not marked by any prominent unconformity, despite the nearly 500 year gap. The layering is parallel to sub-parallel, suggesting that no major, e.g., multi-centennial period of ice recession occurred at that time. However, the layers present are indicative of perhaps decadal-scale ice recession leading to the development of small paraconformities. The stratigraphy in Eisgruben, with its wood-rich sub-parallel layers near the base, corroborates this argument and points towards a positive ice MAMB on centennial scales, with values ranging between 0.8 and 5.8 cm w.e. yr^−1^, interspersed with minor episodes of mass loss.

The next two centuries (800 CE to 1000 CE) saw a drop in MAMB at those two caves, while at Guffert, MAMB was either null or negative. Due to the discontinuous nature of the exposure, we cannot exclude that some of the ice present in Guffert dates from that period. At Eisgruben, this period coincides with the first two of the three large angular unconformities, where a significant amount of ice was lost, altering the geometry of the ice surface. At Bärenloch, there is no evidence for positive MAMB during this period.

The next major change in MAMB occurred at around 1100 CE, when the MAMB rose above 1.0 cm w.e. yr^−1^ at Eisgruben, and a positive MAMB is attested at Guffert, with similar values. At both these sites, the character of the ice changes markedly, transitioning upwards into thicker layers containing far fewer woody macro-remains. At Eisgruben, the clean ice section indicates a positive MAMB until the early 1700s CE (Fig. 3). At Guffert too, the MAMB remained positive for several centuries, ranging between 1 and 4 cm w.e. yr^−1^ (Fig. 3). This time marked the onset of positive MAMB at Hochschneid, at Hundsalm and at Kraterschacht, varying between 1.8 and 2.8 cm w.e. yr^−1^. The oldest parts of the ice body in Großer Naturschacht and Tremml were deposited starting in the 1700s CE, at a time where the records from other caves came to an end. Both cave sections exhibit a minimum MAMB value far exceeding the others, with averages of 5.0 and 13.6 cm w.e. yr^−1^ for Großer Naturschacht and Tremml, respectively (Fig. 3).

Only few mass balance records are available for recent decades in these alpine caves^36,37^, one of them being at Hundsalm ice cave, where accelerating ice loss is observed at the level of *Eisdom*^38^. Using a comparison between old photographs, reports and topometric surveys with our more recent visits, we report an ice mass loss at many of the sites. At Tremml, for instance multi-decadal ice loss amounts to almost a third of the ice volume present. Meanwhile, at Bärenloch, ice has been shown to retreat from the bed-rock walls near the base of the ice, as well as from the top, with a maximum of 10 m of ice lost between 1883 and 2018^34^. At Eisgruben, the topmost 10 m-thick firn deposit has disappeared within four decades of its discovery (Extended Data A.6). The estimated ice loss at Kraterschacht led to a drop in levels of the firn body of almost 20 m in two decades^35^. Snow-level monitoring of Guffert ice cave also showed a drastic drop of the firn surface of almost 3 m between 2019 and 2021.

We therefore report overall negative MAMB during at least the last three decades at most underground ice deposits in this study, especially at low-lying sites.

## Discussion

Previously published radiocarbon chronologies have provided evidence of widespread positive mass balance in alpine caves of the Northern Hemisphere during the Little Ice Age^9,11,13,28^. Other studies have demonstrated that some alpine caves also host significantly older deposits, reaching back before the start of the Common Era^12,27^. Our study provides additional evidence for positive MAMB during the early Common Era, and new evidence for ice growth between 600 to 800 CE at three different field sites. In contrast to previous studies, we have pooled together observations and radiocarbon constraints from eight caves, to draw a more thorough picture of past mass balance dynamics. We are thus able to better detect and discuss the effects of the cave geometry on the volume and stratal organisation of the underground deposits. It is the first time such a comprehensive inter-cave comparison and data integration has been done for ice caves worldwide.

Reconstructing a robust chronology in exposed ice cliffs has proven difficult in many cases before, even when abundant radiocarbon dates are available. Firstly, the availability of a continuously exposed section of ice comprising both the oldest ice layers and the youngest seems to be exceptionally rare in sag-type caves. In our study, we identified two main types of geometries: inclined tunnels (e.g., Eisgruben) and vertical pits (e.g., Hochschneid). Only at Eisgruben can a regularly layered deposit be accessed from top to bottom without interruption. Hitherto published ice cave chronostratigraphies where the complete exposure is accessible include the sites of St. Livres^9^, and A294^12^, which are, like Eisgruben, descending tunnels.

Predominantly vertical geometries are seldom associated with single continuous ice outcrops. At Bärenloch, we were forced to divide the analysis of the record in three different (but overlapping) ice exposures. At Hochschneid, the top-most ice stratigraphy is obscured by seasonal ice formation. In Kraterschacht and Großer Naturschacht, the outcrops are also located near the base of the caves, and the ice sections grade into massive snow with faint to non-existent layering, and little woody material. At Guffert, the two separate sections bracket two periods of positive MAMB with no temporal overlap. The initial study of Hundsalm^13^ initially ran into similar difficulties but nevertheless identified a continuous exposure with dated macro-remains in normal stratigraphic order. Making stratigraphic sense of separate outcrops was equally a challenge at the ice deposit of Strickler Cavern (northern US)^11^. At all these sites, the ice outcrops are discontinuous and the retreat of a crevasse between ice and bedrock allows seasonal snow to fall in, further obscuring the stratigraphy. The ice bodies of vertical pits have a complex geometry dictated by the shape of the host cave. Liquid precipitation, dripwater and rain falling directly onto the ice or firn deposit form holes several metres deep below the firn surface, melting previously deposited snow. Crevasses forming on the side of the ice body during the melting season are also the first to be refilled following new snowfalls. In descending tunnels where most of the ice body is sheltered from the open sky, deposits are less prone to disturbance. Such a geometry thus provides the best chance for preserving a minimally disturbed ice record.

Owing to the lag between the time of wood growth, its transport into the cave, and finally, its inclusion into the ice stratigraphy, the radiocarbon date provides a maximal age for the ice layer. This lag may reach several decades for wood from old trees, but is likely shorter for small twigs or other similarly fragile organic macro-remains, as most samples in this study. Finally, the transport and storage of these organic inclusions within the cave before their embedding within the ice body depends on cave geometry. In the studied sag-type caves, this time is likely short as ice /snow occurs near vertically underneath the respective entrances. Nonetheless, too-old wood detected with the outlier analysis of the age-model is thus interpreted to have been stored in the cave before its incorporation within the ice deposit. Howevern material falling in the caves at times of positive mass balance is likely embedded quickly after being introduced into the cave, eventually appearing as sporadic macro-remains found in clean ice.

As crevasses appear between the ice body and the bedrock walls of the cave, material falling between rock and ice could be reattached to the stratigraphy by the freezing of water or the in-falling of relatively younger snow, though both processes here would lead to a visible change in the stratigraphy, which we specifically avoided in the sampling procedure. As drip-water holes develop over the accumulation surface, organic material and new snow or congelation ice may penetrate deeper in the cave and the ice stratigraphy and bring young material in contact with older ice. At Guffert, the top 5 m of the stratigraphy are affected by such deep drip holes forming seasonally and refilled after the next winter snows. Such a process could cryptically remobilise organic matter and bring samples of various ages into contact, but would be limited to the upper part of the ice body i.e., as far as those seasonal drip water holes develop. The maximum age offset would thus depend on the rate of ice accumulation in the cave, with high accumulation rates minimising the time spent by deposited snow/firn within the part of the ice body vulnerable to the remobilisation process. An example where this process may have taken place in the past is Bärenloch ice cave, specifically for all the samples found in the *deep* and the *middle* section, as the age offset never exceeds 250 years (Fig.B.2), equivalent to a remobilisation over a depth of 1 m given the average MAMB at the time (Fig. 3). Sample BL-C24 however has a large offset of near 600 years with respect to its nearby stratigraphy (Fig.B.2), equivalent to a remobilisation over a depth of ca. 20 m, which is too large for a drip-water hole. Hence, the sample was more likely transported down a crevasse between rock and ice and incorporated by the freezing of drip-water in the cave.

Despite the shortcomings of discontinuous ice exposures, where the exact spatial relationship between parts of the ice sections is not known with high certainty, we can refine the history of past mass balance using the cross-cutting relationship between units of concordant ice layers. Radiometric dating of wood remnants organised in prominent horizons helps constrain past ice mass balance history in two ways. On one hand, prominent layers separating layered ice packages are a marker of past ice ablation^10,27^. In the absence of excessive wood remobilisation, the repeated radiocarbon dating of such layers can provide a minimum estimate of the duration of this interval of mass loss^22^. On the other hand, datable woody remains bracket the maximum duration of ice deposition between two such layers. Consequently, robust age control usually achieved near the deposit bases, where the layering is most conspicuous and stratigraphic principles provide additional constraints on the timing and duration of positive and negative MAMB. Massive, unstratified firn, ubiquitous in the uppermost sections of deposits in sag-type alpine ice caves evades radiocarbon-based chronological control, and their interpretation relies on historical accounts wherever they are available, e.g., at Bärenloch or Hundsalm. At these long-known deposits, it is possible to add additional strong age constraints on the maximum top age of the ice based on old photographs or surveys. At other, more recently discovered sites, we rely on the earliest account of exploration e.g., 2016 CE, for Hochschneid.

An additional difficulty arises from reversals in the radiocarbon calibration curve from 1600 to 1950 CE^39^, spreading the calibrated age probability density over several centuries with, a priori, equal areas underneath the curve. These uncertainties were, however, reduced by Bayesian age modelling, taking advantage of additional knowledge on the stratigraphic position. These difficulties make relatively post 1600 CE ice deposits more challenging to date and robustly constrain using radiocarbon. Nevertheless, in this study, we were able to place further constraints of the temporal pattern of cave glaciation in underground deposits of the European Alps. The onset of the cave glaciation may have occurred at the earliest during the opening of the cave in its current geometry and at the latest, at the time given by the oldest radiocarbon date. In addition, we reconstructed centennial-scale variations in the mass balance of ice caves using well-dated sections of concordant ice units spanning the Common Era.

Past positive or negative mass balance of underground ice likely responds to similar climate variables as surface glaciers, e.g., the timing and amount of past precipitation which controls accumulation, or air temperature, which controls ablation. Although there are key differences between these two members of the cryosphere, for instance the absence of short-wave (solar) radiation in the cave environment, or the relatively small influence of summer air temperatures^40^ in sag-type caves, past periods of glacial advances are likely to have also seen positive MAMB in ice caves. Likewise, periods of reduced ice extent on the surface should coincide with times during which underground ice deposits could disappear entirely or develop large unconformities due to the lowering of the ice surface by melting.

The oldest dated ice section from sag-type caves is located at Eisgruben ice cave, where the thinly layered *Eiszungendom section* provides evidence for cave glaciation as early as ≈ 3700–3400 BCE. Owing to the lack of further age constraints, we conclude that the period spanning 3500–0 BCE did not lead to the complete deglaciation of Eisgruben. Notably, this period of very slightly positive MAMB coincides with a nearly continuous two-millennium-long period of glacier recession in the Swiss Alps^41^. The onset of cave glaciation was coeval with the beginning of a 5000 year-long trend of decreasing summer air temperatures in the Alps^42^.

Glacier readvances have been documented for the Mer de Glace glacier and Great Aletsch glacier from about 600 BCE onwards^43–45^. In the eastern Alps, the Pasterze and Gepatsch glaciers also exceeded their 1940’s extent between 300 and 400 CE 4)^46^. The sections in Bärenloch, Guffert and Eisgruben document an overall positive MAMB during the period from 300 to 400 CE (Fig. 4). In each case, the sub-parallel layers (paraconformities) provide strong evidence of short (annual to decadal) periods of ice loss. In the early Common Era, favourable climatic conditions saw the tongue of the Great Aletsch glacier (Fig. 4)^43^ retreat from an earlier peak at around 700 BCE. For this time period, Sancho et al.^12^ also derived MAMB values of 0.21 cm yr^−1^ between 400 BCE and 60 CE from a Pyrenean ice cave. This rate is comparable with the values derived from coeval sections at Bärenloch, Eisgruben, and Guffert. In the example of the Pyrenean cave, the repeated radiocarbon dating of several layers suggested that paraconformities represent short ablation periods ranging from a few years to a decade, while major angular unconformities range up to multi-centennial scale, such as one long period ranging from 950 BCE to 450 BCE^12^.

The end of the Roman Period in the 5th Century CE heralded a period of hydroclimate instability and summer cooling, captured by tree-ring chronologies in the Alps^47–49^, There is evidence for a Great Aletsch glacier advance from 500 CE to 700 CE, reaching a maximal length just shy of the subsequent LIA maxima (Fig. 4), followed by advances from Pasterze and Gepatsch glaciers^46^. At this time, the sections at Hundsalm, Eisgruben and Bärenloch record an increasingly positive MAMB during that period, ranging between 1 to 5cm w.e. yr^−1^. Positive MAMB was also reported from St-Livres ice cave during the period from 700 CE to 1200 CE.

From the end of the 7th Century CE onwards, tree-ring chronologies suggest that summers were wetter and warmer than average, and hydroclimate variability was reduced until about 1000 CE^47^. The end of this period overlaps with the Medieval Climate Anomaly (MCA, 950 CE to 1250 CE^50^), a period of improved climate in Europe with reduced glacier extents in the Alps in particular^43^. It also coincides with a gap in the mass balance records of Hundsalm and Guffert (Fig. 4), while prominent unconformities are present on the ice cliff of Eisgruben, and the MAMB drops below the section’s average (Fig. 3). At Hundsalm, the MCA is represented by a prominent unconformity. In the St-Livres cave of the Jura Mountains, the MCA was characterised by a significant drop in MAMB between about 1000 CE and 1400 CE, and especially two periods of significant mass loss during the 14th and 15th Centuries CE^9^. The radiocarbon dataset from Hundsalm, Eisgruben, Guffert and Bärenloch highlights periods of ice loss at around 700 CE and 1200 CE, resulting in significant changes to the geometry of the ice surface at those caves; this led to the development of angular unconformities in the ice sections. We conclude that the MCA saw overall negative MAMB in alpine ice caves. It is likely that some ice deposits which could have developed prior to this period were completely lost and some sites temporarily turned into ice-free caves.

The LIA^51^ saw multiple significant advances of glaciers in the Alps^43,52–54^, which rank as the most extensive of the last 11,000 years. This pattern is mirrored in alpine ice caves. The *top sections* in Bärenloch and Guffert, *Steinlawinenschacht* at Eisgruben, as well as the *Iglu section* at Hochschneid provide examples of layered ice successions characterised by successive paraconformities. During the period 1300 CE to 1700 CE, corresponding to the LIA, the record at these three sites indicates that short periods of ice ablation were interspersed within a period of otherwise positive ice mass balance. The much thicker layers, with sparser organic-rich horizons observed at Kraterschacht and Tremml–413 speak of an overwhelmingly positive MAMB during the last centuries of the LIA e.g., 1700 CE to 1850 CE when Europe experienced lower-than-average temperatures, and higher-than-average precipitation^47^. This period was contemporaneous with the maximal extent of the tongues of the Great Aletsch glacier and the Lower Grindelwald glacier^43,55^, suggesting that cave ice and surface ice mass balances respond similarly to a changing hydroclimate.

Since the end of the LIA, widespread retreat has been observed at all glaciers in the Alps^2,41^, a trend accelerating in recent decades^1^. The comparison of cave maps, photographs and reports from Eisgruben, Tremml-Schacht-413 and Kraterschacht corroborate these observations of ongoing ice retreat. The retreat of the ice from surrounding walls allowing access to the stratigraphic sections has led to a significant decrease of underground ice volume at several sites, in line with general findings for ice caves of other parts of Europe^5,7,37^.

In conclusion, we present for the first time a comparative study of the mass balance dynamics at eight caves of the Austrian Alps. Based on 107 radiocarbon dated wood remains trapped in ice, we reconstruct a record of positive and negative mass balances over the past 2500 years. We show evidence of periods with positive MAMB which mirror past glacier length changes in the Alps. The LIA corresponds to the period of widespread positive MAMB, with radiocarbon-bracketed sections spanning at least between 1300 CE to 1850 CE. Where older ice is preserved, we provide evidence of positive MAMB in the first 200 years BCE, as well as from 600 CE to 800 CE. The MAMB values are remarkably coherent, not only between sites in the Alps presented here, but also the Pyrenees^12^ and the Jura Mountains^9^. The periods of positive MAMB in alpine caves also coincide with the reconstructed past glacier advances in the Alps.

We find that all studied alpine caves are experiencing, like alpine glaciers in Europe, an ongoing ice retreat, which puts the survival of these deposits under jeopardy. This loss of underground ice under a changing climate, comparable to the accelerated loss of surface ice in the early 21st Century, may lead to the disappearance of a valuable archive within the next decades, at least with respect to sag-type ice caves. A significant effort towards the full documentation and preservation of such ice deposits by way of coring and geophysical methods is needed if this little-known record of late Holocene climate and environmental variability is to be preserved for future studies. We highlight in particular the need to better constrain the mass balance of sag-type ice caves to gain more robust chronological control and a better understanding of the MAMB response to past warm climate intervals. In this way, we may gauge more quantitatively the vulnerability of underground ice deposits to 21st Century climate change.

## Methods

The caves were surveyed using a calibrated laser disto X2 with an accuracy better than 0.05 m for lengths less than 30 m, and less than 0.5° for azimuth and inclination. Wherever recent disto-based surveys (2016–2018) were already available, only a partial remapping was undertaken, to account for both 1) new discoveries and 2) snow/ice level fluctuations opening new areas of cave hitherto inaccessible. Otherwise, new maps were produced by the authors. Original exploration reports and photographs hosted on the Spelix database (the cadastre of Austrian caves, https://www.spelix.at) were used to assess decadal ice variations at long-known sites.

In each cave, the ice sections were logged according to the layer geometry, ice macroscopic structure and organic content. Firn type ice is recognisable due to its generally isotropic grain-like structure, with equidimensional ice crystals, which are rich in impurities and air bubbles. Firn-derived ice is highly reflective and is distinguished in the field by its opaque and white appearance (Extended Data Fig. A.1). On the other hand, heavily recrystallised ice or congelation ice is characterised by high transparency and strong light absorption, lending a blueish tint compared to firn, leading to darker, but clean layers. The position of selected samples was calculated by between-sample and sample-to-layer tape measurements while the dip of the layers was determined using a calibrated laser disto X2. The height above the base was then calculated by summing the distances between layers. The accuracy of the tape-measure positioning is assessed at 2 cm, as this is the average size of samples encased in the ice. The final accuracy of positioning for samples located near the top of each section scales with the square root of the number of measurements, ranging 9–15 cm for the topmost samples of the Guffert bottom and Eisgruben *Steinlawinenschacht section*s, respectively.

For each cave site, large continuous exposures of layered ice were the primary target of sampling small organic macro-remains. Wood macro-remains found in such exposures fall within two categories. The first concerns samples found sporadically within otherwise clean ice units and are not associated with any other detrital material; such samples are interpreted as having been entrapped within the growing ice body shortly after falling within the cave. The second are organic fragments associated with prominent detrital layers, deposited, remobilised and concentrated during periods of negative mass balance.

Woody macro-fossils clearly embedded in the ice were extracted with the aid of a knife blade, or simply by breaking off and hand-picking of twigs or needle fragments. Samples were chosen depending on their clear embedding within the ice matrix: i.e., they have been revealed by the retreat of the ice exposure (e.g., Fig. A.1B). We established Bayesian-age models by dating many woody samples from a single section and between major unconformities. We interpreted too-old outlier material as stored in-cave before incorporation into the deposit and too-young outlier material preferentially as cryptically remobilised by falling in water drip-holes and to a lesser extent, by being entrained into crevasses between bedrock and ice by newly accumulating ice. Prominent angular unconformities in ice sections were modelled as boundaries in the age models of e.g., Eisgruben (section A.6), where three major boundaries (I, II, III) are included in the model. The age probability density function of these boundaries places periods of negative mass balance in a chronological framework. Additionally, each section thus delimited yields a continuous age-depth relationship, and thus a change of ice height over time, from which MAMB can be calculated. Because such a section may also contain a number of paraconformities (e.g., A.1B) not modelled as boundaries, the value of MAMB is a minimum estimate for that period. Using this age-depth information allows us to better constrain MAMB changes in ice caves at the multi-centennial scale.

The ^13^C/^12^C and ^14^C/^13^C ratios were measured by accelerator mass spectrometry (AMS) at the CHRONO Centre, Queens University Belfast, following acid–alkali pre-treatment and graphitisation procedures^56^. *Ages were calculated according to Stuiver and Polach*^57^ and calibrated using IntCal20^39^, further age modelling was carried out using OxCal 4.4^58^. Conventional radiocarbon ages are reported with a 1σ error (Table S1). Calibrated and modelled ages are reported within a 95.6% confidence interval.

Dated samples for each continuous section were modelled as OxCal P_Sequence^58^. The height from the base of the section (*h*) was computed from sample to sample with a tape measure. Bringing the 2D distributed samples to a 1D height model was achieved by projecting the distance between samples onto a line to the ice perpendicular to the layers. To account for the possibility of material being remobilised and thus out-of-sequence relative to the stratigraphy, outlier treatment is incorporated in the model, using the OxCal Outlier_Model, a Student-t test with 5 degrees of freedom, with prior outlier probability set at 0.05. A first section was thus modelled, and according to the posterior probability of each date being outlier, a new section, omitting detected outliers (whether too-old or too-young samples) was then modelled. The relevant age model definitions are given in the Extended Data B.

Age-depth models based on radiocarbon dating usually exhibit monotonic relationships. In the absence of layer counting, changes of annual mass balance cannot be resolved at any higher resolution than the multi-decadal scale. We used the first derivative of such an age-model function to estimate of the mean annual change in height of the ice body (*h*) during periods spanning several decades to centuries. This value is quoted in other studies as the ice accumulation rate^6,12,20^. The mean annual mass balance over a unit area (MAMB), expressed in kg m^−2^, is closely related to the change in height *h*, expressed in cm yr^−1^. To convert to water-equivalent units^59^, we multiply *h*[L T^−1^] by the dimensionless ratio *k* = ρ_*firn*_/ρ_*water*_ , with a ρ_*firn*_ = 0.87 ± 0.18 g cm^36^.

## Supplementary Information

Supplementary Information 1.

Supplementary Information 2.

## Data Availability

All data generated or analysed during this study are included in this published article and its supplementary information files. OxCal model definitions are given in Extended Data B.
