# Supplementary material for: Multi-centennial mass balance of perennial ice deposits in Alpine caves mirrors the evolution of glaciers during the Late Holocene
Source: Sci Rep. 2022 Jul 5;12:11374. doi: 10.1038/s41598-022-15516-9 (PMC9256607; doi:10.1038/s41598-022-15516-9)
Supplement: Supplementary file 2 — Supplementary Information 2. [file 41598_2022_15516_MOESM2_ESM.pdf]

A Detailed cave and sampling descriptions

A.1 Hundsalm Eis- und Tropfsteinhöhle

Hundsalm Eis- und Tropfsteinhöhle (cadastral code: 1266/1, hereafter Hundsalm), is a cave located in the Brandenberg Alps of Tyrol, opening at an elevation of 1,520 m a.s.l., within a spruce and larch forest[1]. It is 55 m deep, with a deeper ice-free gallery separated from the main ice-bearing cave by an air lock. The cave contains two openings, an upper and a lower entrance, which join in the ice-bearing chamber called Eisdom at a depth of 28 m. Layered firn-derived ice is well exposed along the northern parts of the touristic cave trail, in particular near the deepest ice-bearing section of the cave (Tiefster Punkt).

These parts were sampled in detail previously[1], retrieving 19 individual dates. The ice section situated in the northern sector of the cave[1] has been resampled and a new age-depth model was constructed using five additional radiocarbon-dated samples. In contrast to the more southerly parts of the ice body, this 12 m-wide section is characterised by consistent layering and a normal i.e., upwards younging, stratigraphic progression (Extended Data Fig. B.3).

A.2 Bärenloch Eishöhle

The main entrance of Bärenloch Eishöhle (cadastral code: 1742/54, hereafter Bärenloch), opens at an elevation of 1,518 m a.s.l. with a commanding view over the Gsoll valley. The cave is developed in the Grafensteig limestone[2]. Bärenloch has four entrances, of those, entrance b has the largest opening, and it is proximal to the ice body. The main ice-bearing chamber is oriented roughly North-South. The earliest report of the ice levels in this cave (1883 CE) mentions that the entire floor of this chamber was comprised of mirror-like hard ice with its surface dipping at a very shallow 2° (azimuth: 030°) towards the northern end of the chamber[3]. A crevice leading deeper into the cave was noted but not descended.

At this place, a couple of short drops between rock and ice lead to a succession of small rooms where the ice stratigraphy is well exposed. Compared to the most recent ice level survey carried out in 2018, the size of the crevice has greatly increased, going so far as to allow ample access to the lower parts of the cave. Between the summers of 2018 and 2019 CE, a retreat of about 1 m of the ice wall was noted. In light of the observations and measurements of 1883 CE, we postulate that ice level may have been higher by as much as 10 m than the top of the uppermost ice section in 2021 CE. Bärenloch hosts an ice body of modest dimensions (about 22 m thick) whose geometry splits the main accessible stratigraphy in three sections (*bottom*, *middle*, *top*), each modelled separately.

A visit to the cave during the summer 2020 allowed for the first time access to the lowermost part of the ice body, a small alcove in the ice, whose floor is made of angular cobbles, at a depth of -40 m below the main entrance. There, the ice stratigraphy is made up of 1–5 cm-thick layers containing numerous small organic macro-remains, including twigs, cone fragments and very occasionally small branches (< 10 cm in length). The ice itself has a granular texture and is mostly opaque. The average crystal size is < 1 cm, suggesting a firn origin. The *bottom section* of Bärenloch was sampled there.

The *middle section* is exposed in an alcove located at the foot of a 20 m pit called Eisschacht, covering an area of about 5 × 3 m, where prominent organic-rich layers are present. This section is marked by a prominent transition between two layered ice units, marked by abundant organic material, which cuts across the underlying layered ice (Extended Data Figure A.1c). This angular unconformity separates a first unit where the ice is regularly layered (on average 1–2 cm thickness) and contains many bedding parallel organic remains, from a second unit where the ice is organised in thick decimetre-sized layers with far fewer organic inclusions.

The *top section*, measuring about 15 m high, is exposed on the north-east face of the ice body. On this relatively clean ice section, only few wood fragments are included within sub-horizontal, decimetre-sized layers. The ice is largely white and opaque, with cm-sized crystals indicative of a largely firn-ice origin (Extended Data Figure A.1b). This section is continuously exposed up until the bottom edge of the snow cone of Bärenloch.

A.3 Guffert Eisschacht

Guffert Eisschacht (cadastral code: 1262/9, hereafter Guffert) is located in a shallow valley running NE-SW between the peaks of Guffertspitze (2194 m a.s.l.) to the west and Guffertstein (1963 m a.s.l.) to the east. This 41 m-deep cave is developed entirely in south dipping limestone of the Wetterstein Formation[4]. Entrance a (to the south) opens at an elevation of 1,825 m a.s.l. and has the geometry of a large 10 × 5 m funnel, which collects snow. The ice stratigraphy is particularly well developed in the lowest part of the Eisschacht, where layers are rather coarse. Further progress below the ice body leads to a small ice alcove exhibiting a thin, densely layered ice section with abundant woody macro-remains. The ice stratigraphy of Guffert ice cave is thus split between and a *bottom* and a *top section*.

The deepest, accessible part of the cave (-41 m) is a small 1 m high alcove exposing the contact between the ice body and the underlying cobble-sized scree floor. Dense, laterally continuous, sub-parallel layers range from sub-centimetre to centimetre scale and contain numerous woody macro-remains. The ice is white, with equant crystals about < 1 cm in size (Extended Data Fig. A.1e). This short, condensed *bottom section* of Guffert covers a stratigraphic height of ≈ 1 m, where seven samples were obtained and radiocarbon-dated.

Moving up through the cave, the basal and middle part of the ice pit make an approximately 14 m-tall *top section*. The ice pit is roughly cylindrical, its circumference is almost entirely made of ice. Bedrock outcrops only on the eastern wall of this pit. The cliff which developed opposite of the water inlet from the eastern wall by ice retreat shows a well-developed layered stratigraphy, from which 13 wood samples were obtained (Extended Data Fig.B.5). The water inlet is active from spring to autumn and its spray contributes to the near-cylindrical geometry of the ice pit. The stratigraphy is characterised by thick 40–70 cm layers of opaque, white, isotropic, firn-derived ice. Near the base of this section, faint and smaller-scale layering highlights alternating firn-derived layers with recrystallised ice lenses. . Laterally continuous layers rich in macro-remains dip generally toward the east at an angle ranging 30–50°. The geometry of the ice body in the upper part of the ice pit is heavily influenced by seasonal variations in snow height, which are on the order of ±5 m.

A.4 Großer Naturschacht

Großer Naturschacht (cadastral code: 3741/8, hereafter Naturschacht) is located at the western edge of the Villacher Alpe, close to the Dobratsch summit (2166 m a.s.l.), in a shallow valley called Bärental. The single entrance of Naturschacht opens at an elevation of 1,985 m a.s.l.[5]. The cave is chiefly developed in limestone of the Wetterstein Fomation[6]. Inclined ice layers with prominent woody macro-remains are well developed in this cave, starting from a depth of about 50 m at the *Rutschbahn*, and continuing to the bottom of the cave in the Weißer Dom. Those parts of Naturschacht were the main targets of sampling.

The *Rutschbahn* ice section of Naturschacht is a 15 m long, descending outcrop. The stratigraphy is chiefly comprised of firn-derived ice, opaque and isotropic. Organic-rich layers are few and far between, laterally discontinuous. The ice layers reach up to 1 m thickness and strata are generally inclined at a 40–50° angle towards the north. Along the few apparent wood-rich layers, several conspicuous branches or large twigs partly encased in the ice body are present. Between these layers, ice poor in the macro-remains includes rare twigs or pine cone fragments, as well as lenses of recrystallised ice. Seven wood samples could be taken from this exposure (Extended Data Fig. B.6)

In the Weißer Dom area, another section of ice stratigraphy can be observed. The ice of this section exhibits decimetre-scale layers, markedly darker than above, which has undergone a larger degree of recrystallisation. There, wood fragments are rare, and laterally continuous wood-rich layers are entirely absent. Nevertheless, six wood samples were taken from this *Weißer Dom section*, four of which yielded a modern radiocarbon age.

A.5 Tremml-Schacht-413

Tremml-Schacht-413 (cadastral code: 1744/413, hereafter Tremml) is located in the Hochschwab massif in Styria. This vertical pit, developed in limestone of the Wetterstein Formation[7,8] opens at an elevation of 1,910 m a.s.l. with a first shaft of 100 m to the firn surface. The sloping surface drops about 20 m to a lip, over which it is possible to abseil a further 40 m to the base of the ice deposit. This cliff contains thick layers poor in woody macro-remains. Two radiocarbon samples were retrieved from the base of this approximately 30 m ice section.

The cave was first explored and mapped in 2002. At the time the ice surface position was 75 m below the entrance opening[9]. In 2019, we measured the distance to the ice surface and found it 95 m below the entrance, corresponding to an approximately 20 m drop, and loss of 1500–2000 m^3 of ice volume, about 20–35% of the 2002 ice volume. Given this drop in ice level, the top of the ice body corresponds to ice deposited at the very least prior to 2002.

A.6 Eisgruben Eishöhle

Eisgruben Eishöhle (cadastral code: 1611/39, hereafter Eisgruben) is located in Upper Austria on the western slope of the Sarstein massif, about 700 m southwest of the summit of Hoher Sarstein (1975 m a.s.l.). The cave, opening at an elevation of 1,695 m a.s.l, is developed entirely within well-bedded limestones of the Upper Triassic Dachstein Formation[10]. Eisgruben is an inclined cave with a single entrance containing a spectacular ice deposit, whose volume is estimated at 7×10^3 m^3. The prominent ice layering encountered at the ice cliff of Steinlawinenschacht and further down in Eiszungendom warranted detailed radiocarbon sampling. The original survey carried out in 1982 as well as photographic evidence from that time attest to the presence of a sizeable firn deposit within the entrance chamber. It was necessary to abseil 10 m between the bedrock wall and firn to reach the cave beyond. Survey marks painted onto the cave wall in the 1980s lay some 10 m above a debris talus free of firn in 2021. The first ice was encountered below this (now seasonal) snow cone, where they take the form of a very flat expanse of ice > 50 m^2 called Eislaufplatz.

The Steinlawinenschacht ice cliff of Eisgruben is a 22 m high, overhanging outcrop, where regular, decimetre-scale ice layering is exposed by the retreat of the ice wall (Extended Data Fig A.1a). The ice pit bells out in its lower part, which is characterised by prominent laterally continuous, layers rich in organic macro-remains, dipping at a gentle 20–25° to the NE. Wood-rich layers become scarcer moving up the stratigraphy and disappear entirely after about 4 m height, giving way to clean, regularly spaced ice layers. The ice in Steinlawinenschacht has a mixed origin, with few but prominent white/opaque layers of clear firn origin intercalated with blueish, transparent layers, especially near the top of the ice pit. Firn-derived layers are more common in the vicinity of the wood-rich horizons, where their thickness varies between 10 and 50 cm. Three of the

prominent wood-rich layers occur at major angular unconformities. By rappelling down the *Steinlawinenschacht*, one section could be sampled to the right hand-side (SW) of the outcrop (*SLS-right*). Due to the moderate dip of the layers, a second diagonal transect (*SLS-left*) could be made on the left hand-side (NE) of the outcrop. Five samples were retrieved from this section (Extended Data Fig. B.6b).

A.7 Kraterschacht

Kraterschacht (cadastral code: 1651/24) is a nearly 250 m-deep cave located in the Sengsengebirge of Upper Austria[11]. Its entrance opens at 1530 m a.s.l., and the cave is predominantly vertical, with an entrance pit roughly 100 m deep leading to an elongated chamber hosting about 1.5×10^5 m^3 of ice, by far the largest ice volume in this study. Kraterschacht was discovered in 1990 and surveyed in 1994. Changes of the geometry of the ice deposit hosted in this cave have been observed on several occasions since[12]. The ice body was split shortly before 2003 by a 15 m wide crevasse, preventing access to the *Eiswallschacht section*. The ice level in 2009 had dropped 20 m below a pair of anchor points placed in the cave between 1990 and 1993, now inaccessible. The collapse of a part of the ice body, dated to no later than 2009, led to the deposition of about 1000 m^3 of ice at a depth of 240 m, still observable today. A decrease of about 6% of the total ice volume was reported between 1992 and 2009[12].

A pilot sample extracted in 2009 yielded a ^{14}C age of 886 ± 45 ^{14}C yrs BP[12]. Four more samples were taken during another reconnaissance trip in 2018 (Extended Data Fig. B.7). The main ice wall extends over 50 m in height in the lower part of the cave, with faint, sub-horizontal regular layering in evidence at its base[12].

A.8 Hochschneid Eishöhle

Hochschneid Eishöhle (cadastral code: 1567/172, hereafter Hochschneid) is found a short distance off the Kaiserweg, a well-trodden alpine trail running along the southern fringe of the Höllengebirge, between Hochschneid summit (1752 m a.s.l.) and Helmeskogel (1636 m a.s.l.). This 163 m-deep cave, opening at an elevation of 1,479 m a.s.l. is developed in limestone of the Wetterstein Formation[13] and contains a large snow and ice deposit[14], whose volume is estimated at 4×10^3 m^3. Hochschneid possesses two entrances of markedly different opening diameter and elevation. In practice, it is entrance (a), Nebelmonsterdoline, 40×20 m in diameter, which provides the main pathway for snow input into the cave. Hochschneid hosts the second largest ice volume of the sites under consideration. The lowermost part of the ice body (Iglu) exhibits a clear layered stratigraphy, which was sampled for radiocarbon dating (Extended Data Fig. A.1d).

The *Iglu* outcrop is a ≈ 10 m wide and ≈ 4 m high ice cliff made up of steeply inclined, faint ice strata, separated by sub-parallel horizons rich in woody macro-remains. The ice is generally opaque and made up of isotropic crystals that are strongly reflective and white in appearance. The large-scale layering of the outcrop is emphasised by the layers rich in macro-remains, whose layer thickness ranges between 10 and 70 cm. Faint layering in the sections poor in macro-remains ranges between sub-cm to multi-cm scale. Whilst the layering in the *Iglu section* is near vertical, the examination of the entire ice pit (about 40 m in height) reveals that the layer thickness increases upwards, while the dip decreases markedly, reaching near horizontal values in the Hüttenwirtehalle. In the *Iglu section*, the younging direction is to the left, i.e., from North to South, which agrees with orientation of entrance as major axis.

References

1. Spötl, C., Reimer, P. J. & Luetscher, M. Long-term mass balance of perennial firn and ice in an Alpine cave (Austria): Constraints from radiocarbon-dated wood fragments. *The Holocene* **24**, 165–175, DOI: https://doi.org/10.1177/0959683613515729 (2014).
2. Bryda, G. & van Husen, D. *Geologische Karte der Republik Österreich, 1:50.000 Blatt 101 Eisenerz* (Geologische Bundesanstalt, Wien, 2010).
3. Sahlender, I. Die Erste Durchforschung des "Bärenloches" bei Eisenerz. *Österreichische Touristen-Zeitung* **10**, 112–114 (1883).
4. Spötl, C., Gruber, A., Racine, T. M. F., Scholz, D. & Honiat, C. Karst und Höhlen am Guffert (Nordtirol). *Die Höhle* **72** (2021).
5. Jenatscke, U. Der Naturschacht 3741/8 aus der Villacher Alpe (Kärnten). *Die Höhle* **19**, 58–63 (1968).
6. Anderle, N. Geologische Karte der Republik Österreich, 1:50.000 Blatt 200 Arnoldstein (1977).
7. Bryda, G. *et al. Erläuterungen zu Blatt 101 Eisenerz Geologische Karte der Republik Österreich, 1:50 000* (Geologische Bundesanstalt, Wien, 2013).
8. Plan, L. Speläologische Characterisierung und Analyse des Hochschwab-Plateaus, Steiermark. *Die Höhle* **55**, 19–33 (2004).

9. Plan, L. Neuentdeckte Höhlen in der Hochschwabgruppe (1740) im Jahr 2002. *Höhlenkundliche Mitteilungen des Landesverein für Höhlenkunde Wien und NÖ* **59**, 31–36 (2003).

10. Schäffer, G. *Geologische Karte der Republik Österreich, 1:50.000 Blatt 96 Bad Ischl* (Geologische Bundesanstalt, Wien, 1982).

11. Weißmair, R. Höhleneisbildung aus Schnee und Eisdynamik im Kraterschacht (Sengsengebirge, Oberösterreich). *Die Höhle* **46** **(2)**, 32–37 (1995).

12. Weißmair, R. Eisdatierung und Eis Veränderungen im Kraterschacht (1651/24, Sengsengebirge, Oberösterreich) zwischen 1992 und 2009. *Die Höhle* **62** **(1-4)**, 27–30 (2011).

13. Egger, H. *Geologische Karte der Republik Österreich, 1:50.000 Blatt 66 Gmunden* (Geologische Bundesanstalt, Wien, 1996).

14. Wielander, B., Allhuter, D. & Spötl, C. Neue Ergebnisse der Höhlenforschung im östlichen Höllengebirge. *Die Höhle* **70**, 49–53 (2019).

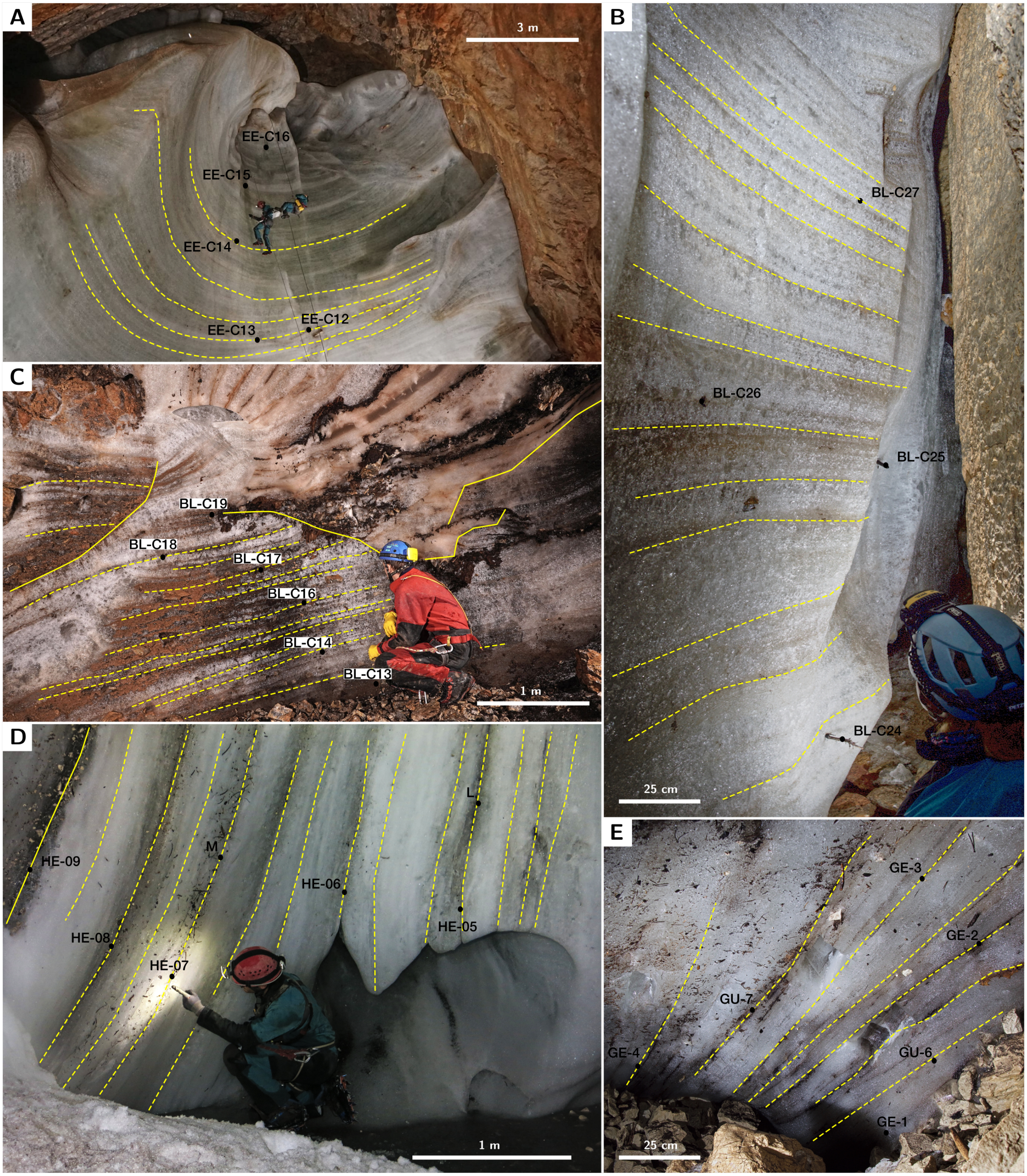

Figure A.1. Selected ice exposures from the study sites. a) *Steinlawinenschacht section* in Eisgruben, photo: C. Spötl. b) Mosaic of the newly exposed Bärenloch *top section*, photo: T. Racine. c) The *top section* in Bärenloch, photo: C. Spötl. d) Steeply dipping ice strata in the *Iglu section* of Hochschneid, photo: R. Fellinger. e) The *bottom section* of Guffert, photo: T. Racine. Solid lines denote major angular unconformities corresponding to multi-decadal (or longer) ice loss. Dashed yellow lines delineate intra-unit bedding and correspond to paraconformities, reflecting intervals of multi-annual ice loss. Not all are shown for clarity.

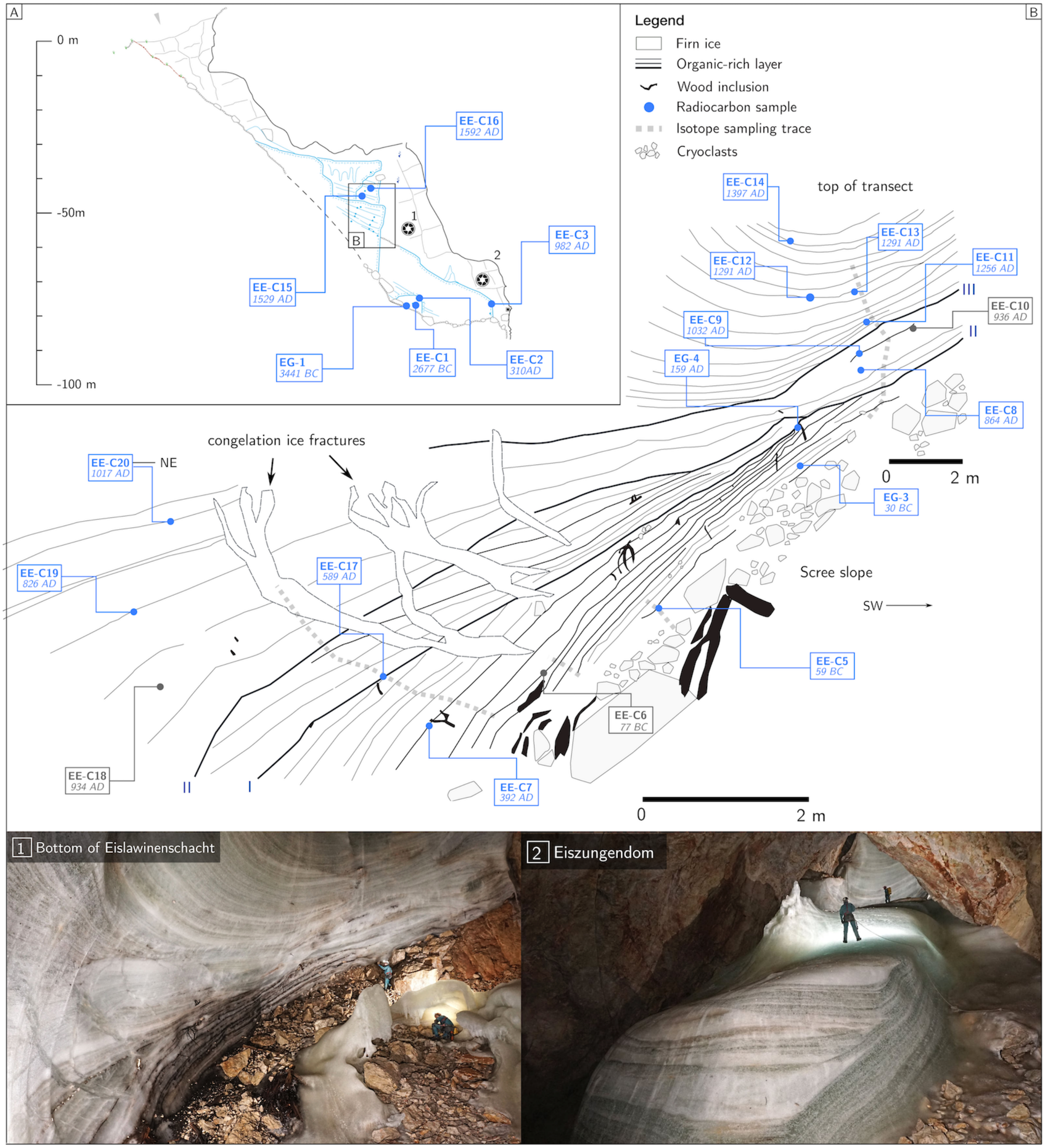

Figure A.2. Survey and sketch of a typical ice exposure. A) Extended elevation view of Eisgruben Eishöhle. B) Scaled sketch of the sampling scheme of the *Steinlawinenschacht section* in Eisgruben. Photographs: C. Spötl

B Age models

In this section, we provide the OxCal model definitions which were used to build the age models. Each *section* is modelled as an OxCal `P_Sequence`.

B.1 Hundsalm

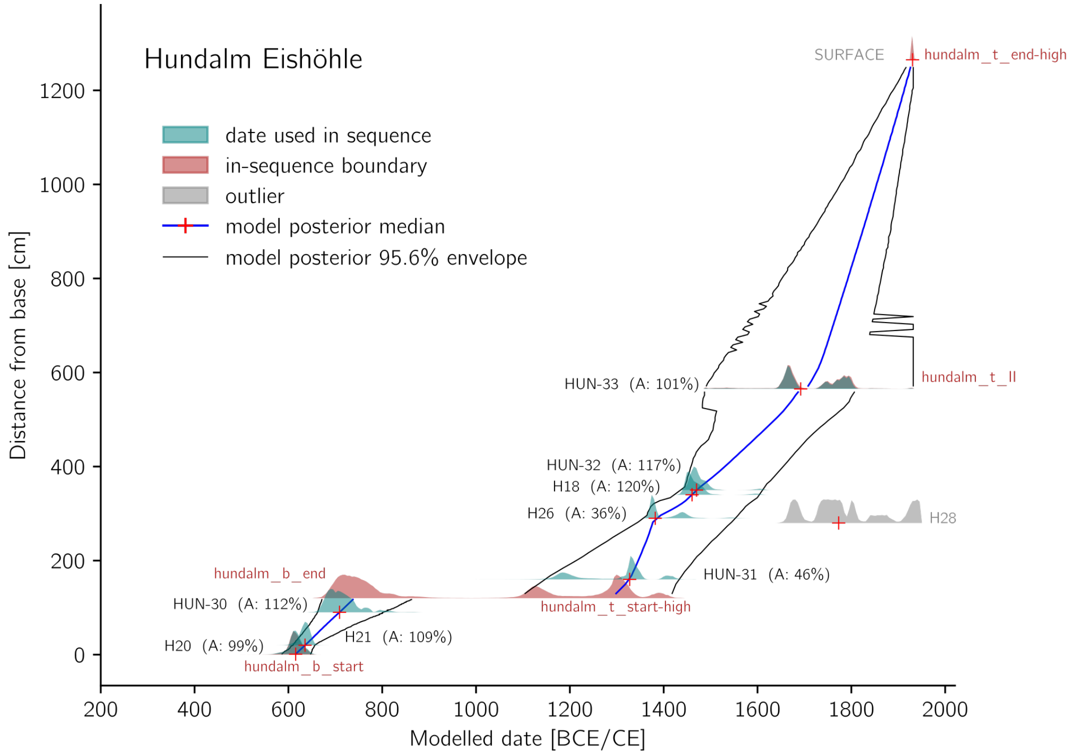

Figure B.3. Age model for Hundsalm

```
Plot()
{
 Outlier_Model("General",T(5),U(0,4),"t");
 P_Sequence("hundalm-low", 1,0.1,U(-2,2))
 {
  Boundary("start-low")
  {
   z=0;
  };
  R_Date("H20",1419,30)
  {
   z=0;
   Outlier(0.05);
  };
  R_Date("H21",1452,30)
  {
   z=20;
   Outlier(0.05);
  };
  R_Date("HUN-30",1258,28)
  {
   z=90;
   Outlier(0.05);
  };
  Boundary("end-low")
  {
   z=120;
  };
```

```
 };
 P_Sequence("hundalm-high", 1,0.1,U(-2,2))
 {
  Boundary("start-high")
  {
   z=120;
  };
  R_Date("HUN-31",549,26)
  {
   z=160;
   Outlier(0.05);
  };
  R_Date("H26",688,22)
  {
   z=290;
   Outlier(0.05);
  };
  R_Date("H18",402,29)
  {
   z=340;
   Outlier(0.05);
  };
  R_Date("HUN-32",411,29)
  {
   z=350;
   Outlier(0.05);
  };
  R_Date("HUN-33",203,25)
  {
   z=565;
   Outlier(0.05);
  };
  Boundary("II")
  {
   z=566;
  };
  Date("SURFACE",N(AD(1930,10)))
  {
   z=1265;
  };
  Boundary("end-high")
  {
   z=1265;
  };
 };
 R_Date("H28",172,29)
 {
  z=280;
 };
};
```

B.2 Bärenloch

B.2.1 Top section

```
Plot()
{
 Outlier_Model("General",T(5),U(0,4),"t");
 P_Sequence("Baerenloch_t",1,0.2,U(-2,2))
 {
  Boundary('start');
  R_Date("BL-C10",906,20)
  {
   z=750;
   Outlier(0.05);
  };
  R_Date("BL-C11",781,20)
  {
   z=925;
   Outlier(0.05);
  };
  R_Date("BL-C26",354,29)
  {
   z=947;
   Outlier(0.05);
  };
  R_Date("BL-C12",310,20)
  {
   z=1009;
   Outlier(0.05);
  };
  R_Date("BL-C27",368,31)
  {
   z=1028;
   Outlier(0.05);
  };
  R_Date("BL-C28",220,25)
  {
   z=2050;
   Outlier(0.05);
  };
  Date("SURF",N(AD(1883,5)))
  {
   z=2550;
   Outlier(0.05);
  };
  Boundary('end');
 };
 R_Date("BL-C24",151,34)
 {
  z=824;
 };
};
```

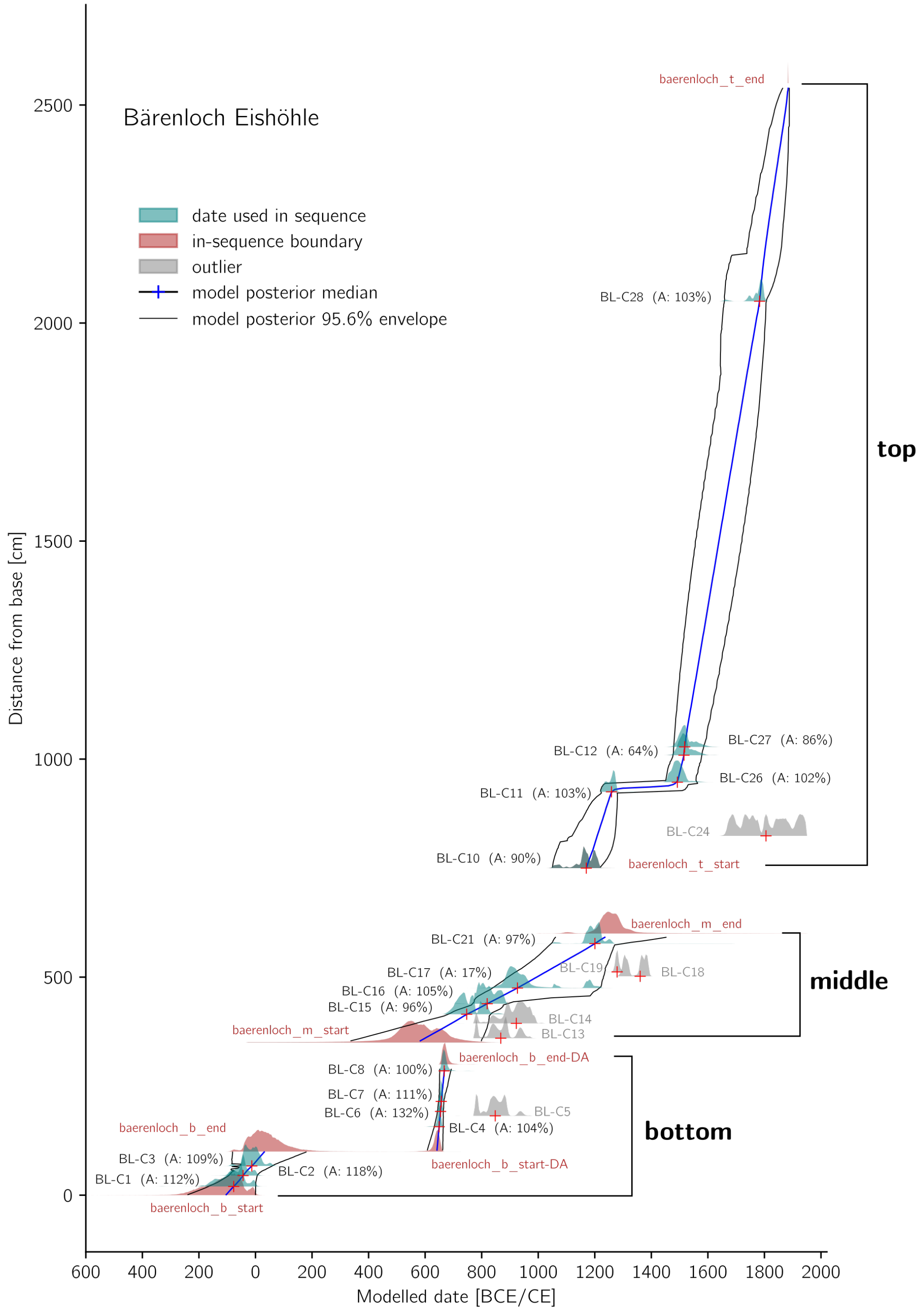

Figure B.4. Age model for Bärenloch

B.2.2 Middle section

```
Plot()
{
 Outlier_Model("General",T(5),U(0,4),"t");
 P_Sequence("Baerenloch_m",1,0.2,U(-2,2))
 {
  Boundary("start")
  {
   z=345;
  };
  R_Date("BL-C13",1167,20)
  {
   z=355;
   Outlier(0.05);
  };
  R_Date("BL-C14",1141,31)
  {
   z=389;
   Outlier(0.05);
  };
  R_Date("BL-C17",851,19)
  {
   z=470;
   Outlier(0.05);
  };
  R_Date("BL-C19",730,20)
  {
   z=507;
   Outlier(0.05);
  };
  Boundary("end")
  {
   z=645;
  };
 };
 R_Date("BL-C15",1264,27)
 {
  z=410;
 };
 R_Date("BL-C16",1207,20)
 {
  z=434;
 };
 R_Date("BL-C18",642,20)
 {
  z=497;
 };
 R_Date("BL-C21",859,24)
 {
  z=572;
 };
};
```

B.2.3 Bottom section

```
Plot()
{
 Outlier_Model("General",T(5),U(0,4),"t");
 P_Sequence("Baerenloch_b",1,0.5,U(-2,2))
 {
  Boundary("start-b")
  {
   z=0;
  };
  R_Date("BL-C1",2080,37)
  {
   z=20;
   Outlier(0.05);
  };
  R_Date("BL-C2",2049,25)
  {
   z=45;
   Outlier(0.05);
  };
  R_Date("BL-C3",2026,23)
  {
   z=67;
   Outlier(0.05);
  };
  Boundary("end-b")
  {
   z=100;
  };
 };
 P_Sequence("Baerenloch_DA",1,0.2,U(-2,2))
 {
  Boundary("start-DA")
  {
   z=100;
  };
  R_Date("BL-C4",1375,25)
  {
   z=157;
   Outlier(0.05);
  };
  R_Date("BL-C6",1390,21)
  {
   z=192;
   Outlier(0.05);
  };
  R_Date("BL-C7",1387,21)
  {
   z=215;
   Outlier(0.05);
  };
  R_Date("BL-C8",1313,24)
  {
   z=285;
   Outlier(0.05);
  };
  Boundary("end-DA")
  {
   z=300;
  };
 };
 R_Date("BL-C5",1179,19)
 {
  z=182;
 };
};
```

B.3 Guffert

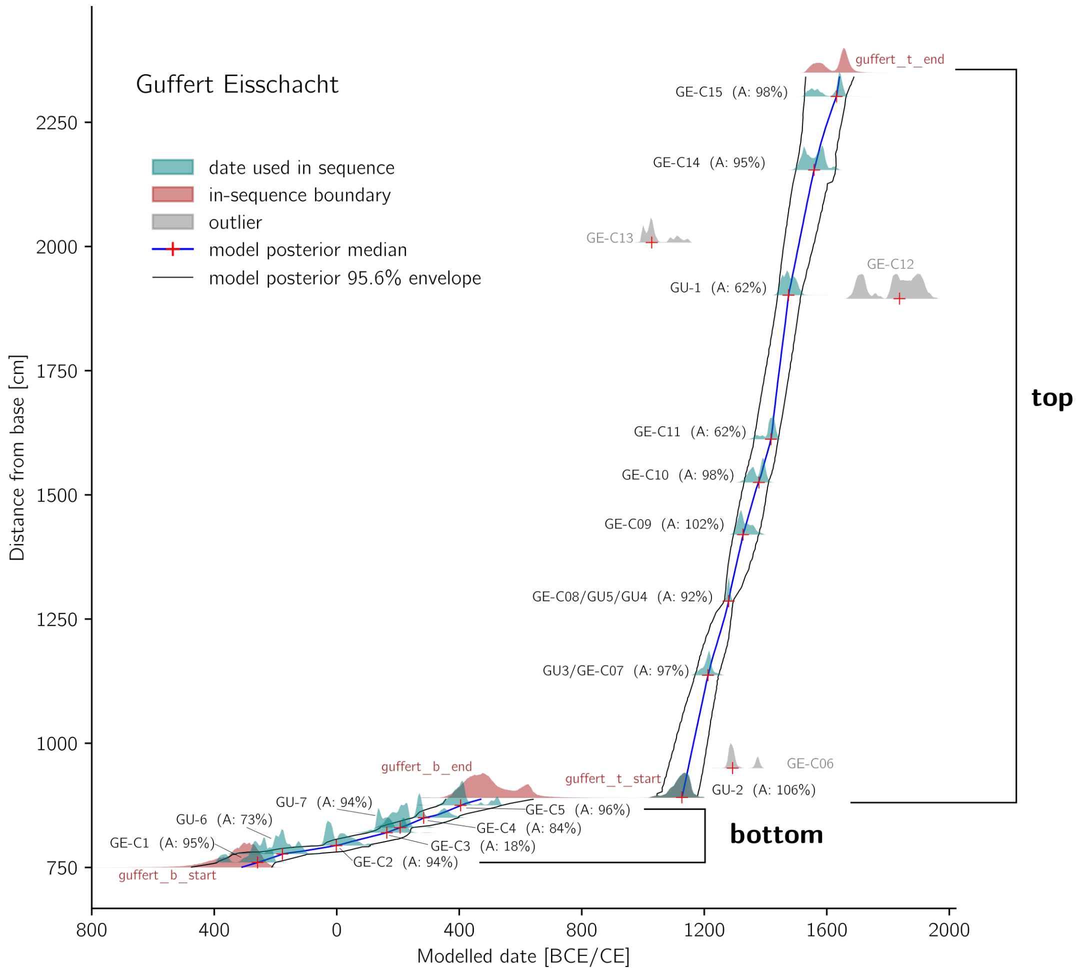

Figure B.5. Age model for Guffert

B.3.1 Top section

```
Plot()
{
 Outlier_Model("General",T(5),U(0,4),"t");
 P_Sequence("guffert_t",1,0.2,U(-2,2))
 {
  Boundary("start")
  {
   z=140;
  };
  R_Date("GU-2",962,35)
  {
   z=141;
   Outlier(0.05);
  };
  R_Combine("GU3/GE-C07")
  {
   R_Date("GU3",785,45);
   R_Date("GE-C07",885,25);
   z=387;
   Outlier(0.05);
  };
  R_Combine("GE-C08/GU5/GU4")
  {
   R_Date("GE-C08",721,25);
```

```
   R_Date("GU5",743,23);
   R_Date("GU4",711,22);
   z=536;
   Outlier(0.05);
  };
  R_Date("GE-C09",628,34)
  {
   z=670;
   Outlier(0.05);
  };
  R_Date("GE-C10",601,31)
  {
   z=775;
   Outlier(0.05);
  };
  R_Date("GE-C11",488,21)
  {
   z=862;
   Outlier(0.05);
  };
  R_Date("GU-1",431,29)
  {
   z=1152;
   Outlier(0.05);
  };
  R_Date("GE-C14",314,19)
  {
   z=1404;
   Outlier(0.05);
  };
  R_Date("GE-C15",276,24)
  {
   z=1552;
   Outlier(0.05);
  };
  Boundary("end")
  {
   z=1600;
  };
 };
 R_Date("GE-C06",698,29)
 {
  z=200;
 };
 R_Date("GE-C12",95,38)
 {
  z=1145;
 };
 R_Date("GE-C13",1007,28)
 {
  z=1258;
 };
};
```

B.3.2 Bottom section

```
Plot()
{
 Outlier_Model("General",T(5),U(0,4),"t");
 P_Sequence("guffert_b",1,1,U(-2,2))
 {
  Boundary("start")
  {
   z=0;
  };
  R_Date("GE-C1",2261,30)
  {
   z=10;
   Outlier(0.05);
  };
  R_Date("GU-6",2178,26)
  {
   z=27;
   Outlier(0.05);
  };
  R_Date("GE-C2",1982,28)
  {
   z=44;
   Outlier(0.05);
  };
  R_Date("GE-C3",1793,30)
  {
   z=70;
   Outlier(0.05);
  };
  R_Date("GU-7",1866,25)
  {
   z=80;
   Outlier(0.05);
  };
  R_Date("GE-C4",1693,30)
  {
   z=100;
   Outlier(0.05);
  };
  R_Date("GE-C5",1674,30)
  {
   z=125;
   Outlier(0.05);
  };
  Boundary("end")
  {
   z=140;
  };
 };
};
```

B.4 Großer Naturschacht

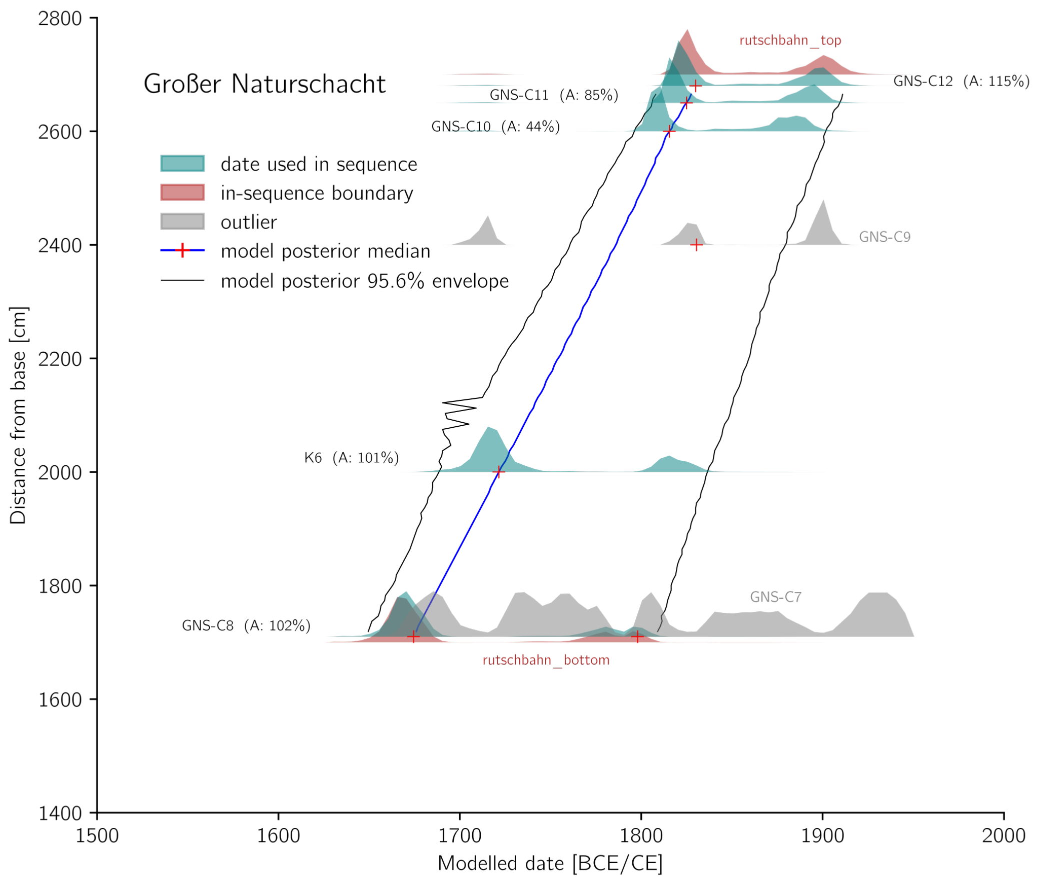

Figure B.6. Age model for Großer Naturschacht

```
Plot()
{
 Outlier_Model("General",T(5),U(0,4),"t"),
 P_Sequence("Eisdom",1,0.5,U(-2,2))
 {
  Boundary("eisdom_bottom")
  {
   z=0;
  };
  R_Date("GNS-C4", 239, 21)
  {
   z=145;
   Outlier(0.05);
  };
  R_Date("GNS-C6", 155, 24)
  {
   z=155;
   Outlier(0.05);
  };
  Boundary("eisdom_top")
  {
   z=170;
  };
 };
 P_Sequence("Rutschbahn",1,0.1,U(-2,2))
 {
  Boundary("rutschbahn_bottom")
```

```
  {
   z=1700;
  };
  R_Date("GNS-C8", 191, 20)
  {
   z=1710;
   Outlier(0.05);
  };
  R_Date("K6", 107,24)
  {
   z=2000;
   Outlier(0.05);
  };
  R_Date("GNS-C10", 166, 20)
  {
   z=2600;
   Outlier(0.05);
  };
  R_Date("GNS-C11", 65, 23)
  {
   z=2650;
   Outlier(0.05);
  };
  R_Date("GNS-C12", 83, 22)
  {
   z=2680;
   Outlier(0.05);
  };
  Boundary("rutschbahn_top")
  {
   z=2700;
  };
 };
 R_Date("GNS-C7", 155, 24)
 {
  z=1710;
 };
 R_Date("GNS-C9", 20, 19)
 {
  z=2400;
 };
};
```

B.5 Tremml-Schacht-413

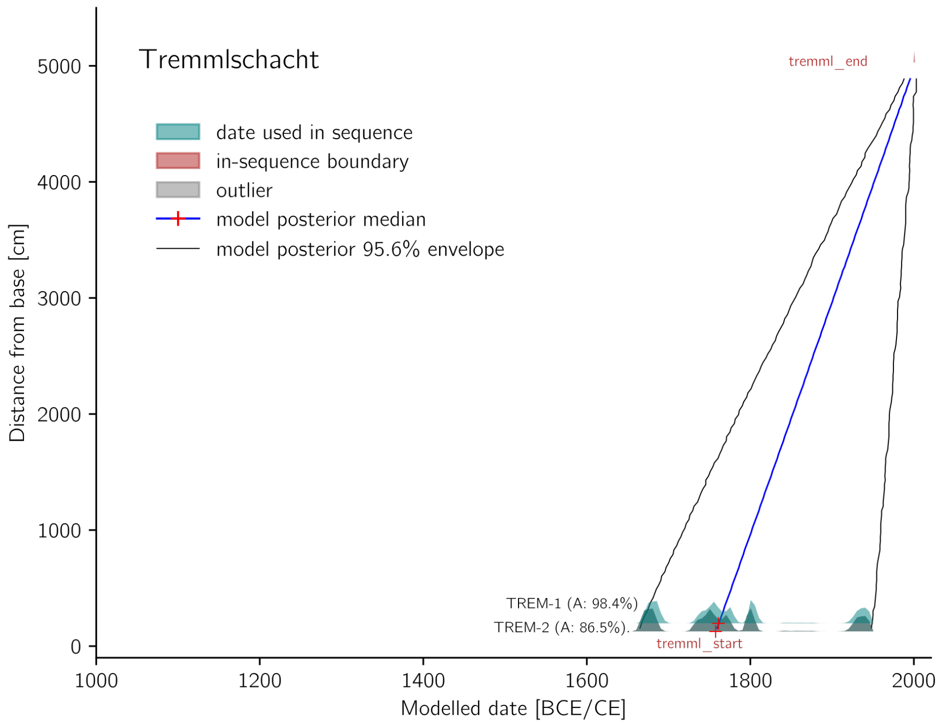

Figure B.7. Age model for Tremml-Schacht-413

```
Plot()
{
 P_Sequence("Tremml", 1, 0.025, U(-2,2))
 {
  Boundary(start);
  R_Date("TREM-2", 199, 24)
  {
   z=120;
  };
  R_Date("TREM-1", 149, 21)
  {
   z=190;
  };
  Date("SURF", N(AD(2000,2)))
  {
   z=5000;
  };
  Boundary(end);
 };
};
```

B.6 Eisgruben

B.6.1 Eiszungendom sequence

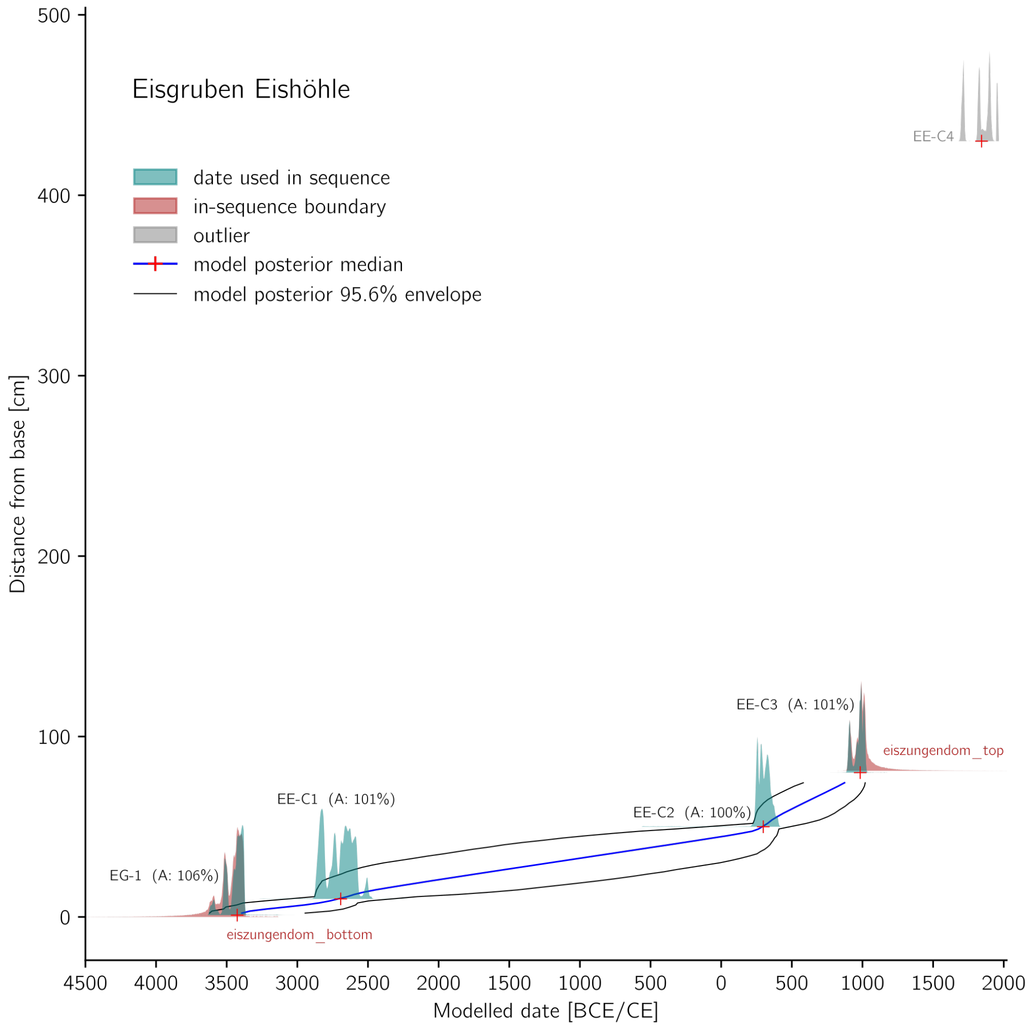

Figure B.6a. Age model for the *Eiszungendom section* of Eisgruben

```
Plot()
{
 Outlier_Model("General",T(5),U(0,4),"t");
 P_Sequence("Eiszungendom",1,0.5,U(-2,2))
 {
  Boundary("bottom")
  {
   z =0;
  };
  R_Date("EG-1",4703,29)
  {
   z = 1;
   Outlier(0.05);
  };
  R_Date("EE-C1",4105,40)
  {
   z = 10;
   Outlier(0.05);
  };
  R_Date("EE-C2",1750,28)
```

```
    {
     z = 50;
     Outlier(0.05);
    };
    R_Date("EE-C3",1072,25)
    {
     z = 80;
     Outlier(0.05);
    };
    Boundary("top")
    {
     z =81;
    };
   };
   R_Date("EE-C4",51,27)
   {
    z = 430;
   };
  };
```

B.6.2 Steinlawinenschacht sequence

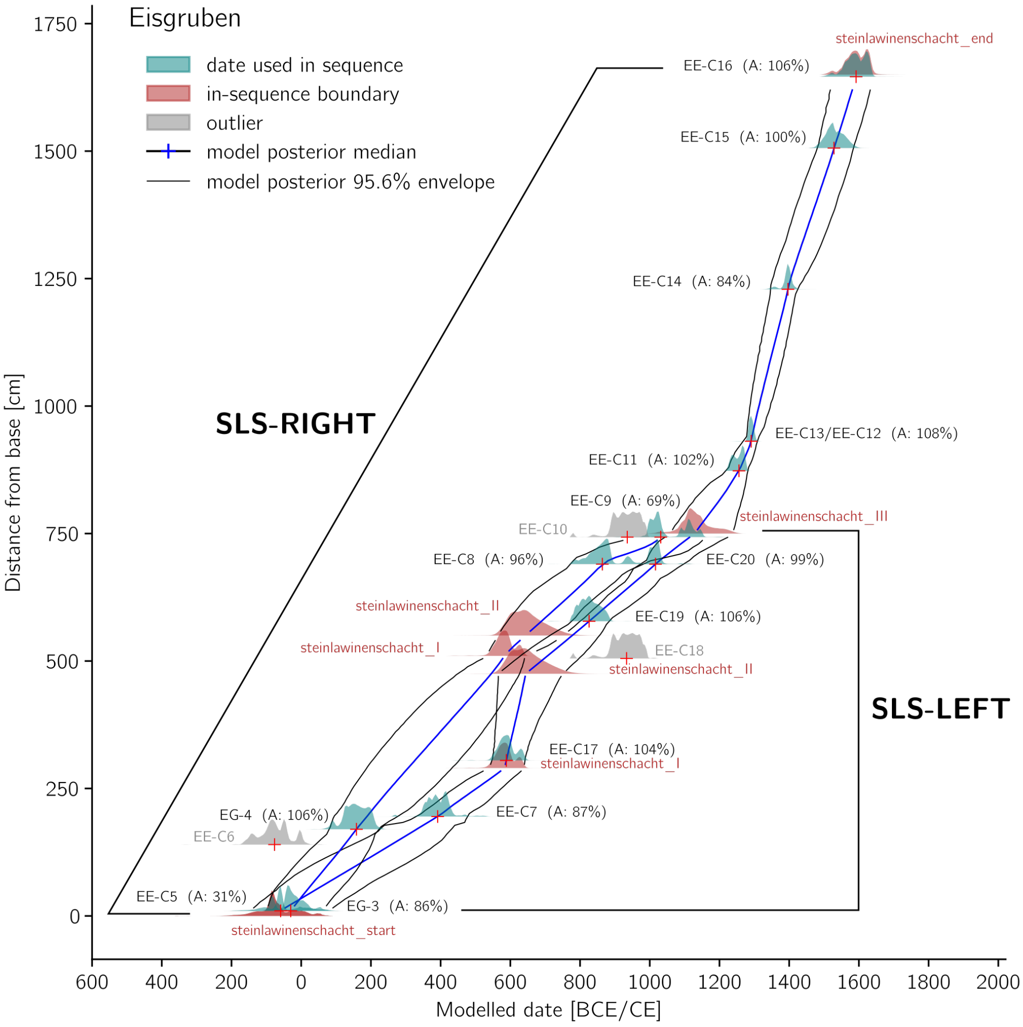

Figure B.6b. Age model for the *Steinlawinenschacht section* of Eisgruben

```
 Plot()
{
 Outlier_Model("General",T(5),U(0,4),"t");
 P_Sequence("SLS-left",1,0.1,U(-2,2))
 {
  Boundary("bottom")
  {
   z=0;
  };
  R_Date("EE-C5",2148,27)
  {
   z=10;
   Outlier(0.05);
  };
  R_Date("EE-C7",1646,28)
  {
   z=195;
   Outlier(0.05);
  };
  Boundary("I")
  {
   z=290;
  };
  R_Date("EE-C17",1492,30)
  {
   z=305;
   Outlier(0.05);
  };
  Boundary("II")
  {
   z=475;
  };
  R_Date("EE-C19",1203,22)
  {
   z=578;
   Outlier(0.05);
  };
  R_Date("EE-C20",1028,24)
  {
   z=690;
   Outlier(0.05);
  };
  Boundary("III")
  {
   z=750;
  };
 };
 P_Sequence("SLS-right",1,0.1,U(-2,2))
 {
  Boundary("=bottom")
  {
   z=0;
  };
  R_Date("EG-3",2019,23)
  {
   z=10;
   Outlier(0.05);
  };
  R_Date("EG-4",1894,24)
  {
   z=170;
   Outlier(0.05);
  };
  Boundary("=I")
  {
   z=510;
  };
  Boundary("=II")
  {
   z=550;
```

```
  };
  R_Date("EE-C8",1196,26)
  {
   z=690;
   Outlier(0.05);
  };
  R_Date("EE-C9",1025,30)
  {
   z=743;
   Outlier(0.05);
  };
  Boundary("=III")
  {
   z=750;
  };
  R_Date("EE-C11",776,21)
  {
   z=873;
   Outlier(0.05);
  };
  R_Combine("EE-C13/EE-C12")
  {
   R_Date("EE-C13",650,23);
   R_Date("EE-C12",704,24);
   z=931;
   Outlier(0.05);
  };
  R_Date("EE-C14",592,27)
  {
   z=1229;
   Outlier(0.05);
  };
  R_Date("EE-C15",324,23)
  {
   z=1506;
   Outlier(0.05);
  };
  R_Date("EE-C16",339,21)
  {
   z=1646;
   Outlier(0.05);
  };
  Boundary("top")
  {
   z = 1650;
  };
 };
 R_Date("EE-C10",1124,26)
 {
  z=743;
 };
 R_Date("EE-C18",1127,27)
 {
  z=505;
 };
 R_Date("EE-C6",2067,25)
 {
  z=140;
 };
};
```

B.7 Kraterschacht

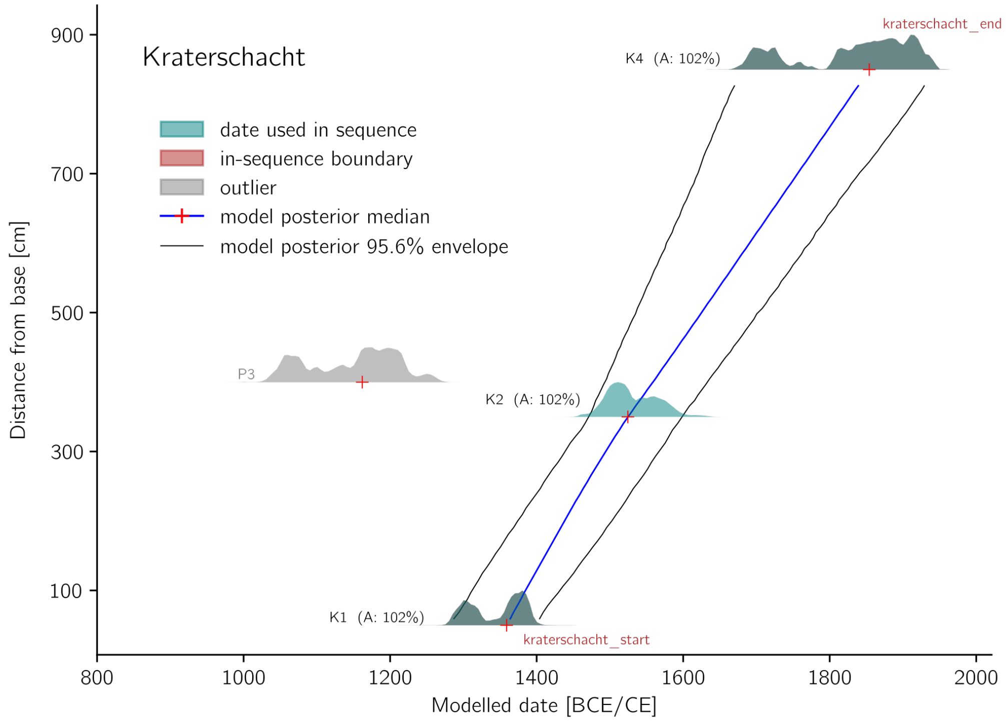

Figure B.7. Age model for Kraterschacht

```
Plot()
 {
  Outlier_Model("General",T(5),U(0,4),"t");
  P_Sequence("Eiswallschacht",1,0.1,U(-2,2))
  {
   Boundary('start');
   R_Date("K1", 650, 34)
   {
    z=50;
    Outlier(0.05);
   },
   R_Date("K2", 336, 30)
   {
    z=350;
    Outlier(0.05);
   };
   R_Date("K4", 120, 28)
   {
    z=850;
    Outlier(0.05);
   };
   Boundary('end');
  };
  R_Date("P3", 886, 45)
  {
   z=400;
  };
 };
```

B.8 Hochschneid

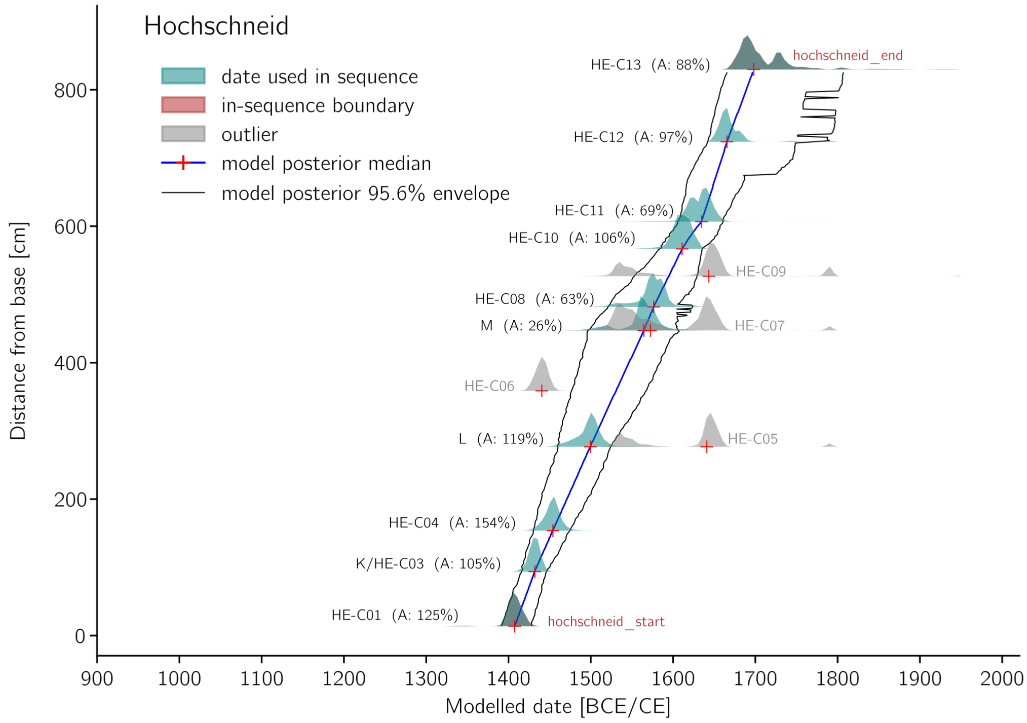

Figure B.8. Age model for Hochschneid

```
Plot( )
{
 Outlier_Model("General",T(5),U(0,4),"t");
 P_Sequence("Hochschneid",1,0.5,U(-2,2))
 {
  Boundary('start')
  {
   z=4;
  };
  R_Date("HE-C01",541,26)
  {
   z=15;
   Outlier(0.05);
  };
  R_Combine("K/HE-C03")
  {
    R_Date("K",509,27);
    R_Date("HE-C03",487,27);
    z=92;
    Outlier(0.05);
  };
  R_Date("HE-C04",426,40)
  {
   z=157;
   Outlier(0.05);
  };
  R_Date("L",366,31)
  {
   z=277;
   Outlier(0.05);
  };
  R_Date("M",387,28)
  {
```

```
   z=427;
   Outlier(0.05);
  };
  R_Date("HE-C08",298,21)
  {
   z=462;
   Outlier(0.05);
  };
  R_Date("HE-C10",364,19)
  {
   z=547;
   Outlier(0.05);
  };
  R_Date("HE-C11",257,28)
  {
   z=587;
   Outlier(0.05);
  };
  R_Date("HE-C12",215,21)
  {
   z=704;
   Outlier(0.05);
  };
  R_Date("HE-C13",158,22)
  {
   z=810;
   Outlier(0.05);
  };
  Boundary('end')
  {
   z=820;
  };
 };

 R_Date("HE-C05",271,20)
 {
  z=277;
 };
 R_Date("HE-C06",460,19)
 {
  z=342;
 };
 R_Date("HE-C07",281,27)
 {
  z=427;
 };
 R_Date("HE-C09",265,25)
 {
  z=507;
 };
};
```

C Radiocarbon calibration results

Table S1. Summary of radiocarbon dates for the studied caves. Asterisks (*) denote samples excluded from the age-depth model of their respective section.

	Lab code	Event	Conventional ^{14}C age BP	Calibrated ^{14}C age range (2σ)
Hundsalm				
	UBA-35896	HUN-31	549 ± 26	1144 CE (32.8%) 1266 CE<br>1294 CE (52.6%) 1361 CE<br>1391 CE (10.1%) 1432 CE
	UBA-20889	H26	688 ± 22	1360 CE (93.4%) 1471 CE<br>1533 CE (2.0%) 1552 CE
	UBA-21456	H18	402 ± 29	1434 CE (94.1%) 1500 CE<br>1592 CE (1.3%) 1601 CE
	UBA-35897	HUN-32	411 ± 29	1441 CE (93.8%) 1508 CE<br>1599 CE (1.7%) 1610 CE
	UBA-35898	HUN-33	203 ± 25	1486 CE (0.4%) 1493 CE<br>1504 CE (2.3%) 1552 CE<br>1636 CE (44.7%) 1694 CE<br>1725 CE (48.0%) 1812 CE
	UBA-20459	H28*	172 ± 29	1659 CE (18.0%) 1700 CE<br>1721 CE (48.6%) 1815 CE<br>1834 CE (10.3%) 1886 CE<br>1909 CE (18.6%) 1948 CE
	UBA-20561	H20	1419 ± 30	586 CE (95.4%) 647 CE
	UBA-20710	H21	1452 ± 30	610 CE (95.4%) 655 CE
	UBA-35895	HUN-30	1258 ± 28	662 CE (94.1%) 777 CE<br>791 CE (1.4%) 801 CE
Bärenloch				
	UBA-43983	BL-C15	1264 ± 27	680 CE (73.0%) 779 CE<br>786 CE (22.4%) 830 CE
	UBA-43984	BL-C16	1207 ± 20	774 CE (95.4%) 878 CE
	UBA-43985	BL-C17	851 ± 19	840 CE (79.3%) 1009 CE<br>1050 CE (3.5%) 1077 CE<br>1160 CE (12.6%) 1220 CE
	UBA-43424	BL-C21	859 ± 24	1046 CE (2.7%) 1084 CE<br>1130 CE (0.1%) 1138 CE<br>1148 CE (92.6%) 1272 CE
	UBA-43982	BL-C13	1167 ± 20	772 CE (14.1%) 792 CE<br>804 CE (0.7%) 810 CE 818 CE<br>(61.9%) 900 CE 918 CE<br>(18.7%) 960 CE
	UBA-43422	BL-C14	1141 ± 31	775 CE (4.3%) 788 CE<br>828 CE (9.4%) 861 CE 868 CE<br>(81.7%) 990 CE
	UBA-43986	BL-C18	642 ± 20	1290 CE (40.4%) 1325 CE<br>1352 CE (55.0%) 1394 CE
	UBA-43423	BL-C19	730 ± 20	1264 CE (95.4%) 1296 CE
	UBA-43415	BL-C1	2080 ± 37	168 BCE (88.9%) 31 BCE<br>25 BCE (6.5%) 1 BCE
	UBA-43417	BL-C2	2049 ± 25	96 BCE (95.4%) 6 CE

	UBA-43977	BL-C3	2026 ± 23	86 BCE (0.8%) 80 BCE 55 BCE (91.2%) 34 CE 40 CE (3.5%) 58 CE
	UBA-43418	BL-C4	1375 ± 25	632 CE (95.4%) 664 CE
	UBA-43979	BL-C6	1390 ± 21	643 CE (95.4%) 664 CE
	UBA-43419	BL-C7	1387 ± 21	648 CE (95.4%) 666 CE
	UBA-43980	BL-C8	1313 ± 24	651 CE (95.4%) 690 CE
	UBA-43978	BL-C5	1179 ± 19	772 CE (91.6%) 894 CE 928 CE (3.9%) 944 CE
	UBA-43420	BL-C10	906 ± 20	1050 CE (8.5%) 1083 CE 1098 CE (0.3%) 1101 CE 1120 CE (86.6%) 1219 CE
	UBA-43981	BL-C11	781 ± 20	1226 CE (95.4%) 1278 CE
	UBA-46336	BL-C26	354 ± 29	1449 CE (95.4%) 1560 CE
	UBA-43421	BL-C12	310 ± 20	1477 CE (95.4%) 1576 CE
	UBA-46337	BL-C27	368 ± 31	1478 CE (95.4%) 1586 CE
	UBA-46338	BL-C28	220 ± 25	1658 CE (2.5%) 1670 CE 1733 CE (93.0%) 1805 CE
	UBA-46335	BL-C24	151 ± 34	1666 CE (43.2%) 1783 CE 1795 CE (34.4%) 1896 CE 1902 CE (17.9%) 1947 CE
Guffert				
	UBA-20525	GU-2	962 ± 35	1044 CE (0.9%) 1051 CE 1061 CE (94.5%) 1176 CE
	UBA-35771	GU-3	785 ± 45	1170 CE (95.4%) 1244 CE
	UBA-41856	GE-C07	885 ± 25	1170 CE (95.4%) 1244 CE
	UBA-35772	GU4	711 ± 22	1267 CE (95.4%) 1292 CE
	UBA-35773	GU5	743 ± 23	1267 CE (95.4%) 1292 CE
	UBA-41857	GE-C08	721 ± 25	1267 CE (95.4%) 1292 CE
	UBA-41858	GE-C09	628 ± 34	1296 CE (95.4%) 1380 CE
	UBA-41859	GE-C10	601 ± 31	1328 CE (95.4%) 1410 CE
	UBA-41860	GE-C11	488 ± 21	1362 CE (95.4%) 1442 CE
	UBA-20251	GU-1	962 ± 35	1440 CE (95.4%) 1515 CE
	UBA-42491	GE-C14	314 ± 19	1500 CE (93.5%) 1604 CE 1618 CE (1.9%) 1628 CE
	UBA-42492	GE-C15	276 ± 24	1528 CE (40.1%) 1591 CE 1620 CE (55.4%) 1662 CE
	UBA-41855	GE-C06*	698 ± 29	1268 CE (73.1%) 1311 CE 1361 CE (22.3%) 1387 CE
	UBA-42489	GE-C12*	95 ± 38	1680 CE (26.3%) 1740 CE 1752 CE (1.4%) 1763 CE 1800 CE (67.7%) 1940 CE
	UBA-42490	GE-C13*	1007 ± 28	990 CE (69.5%) 1049 CE 1081 CE (25.9%) 1151 CE
	UBA-41850	GE-C1	2261 ± 30	390 BCE (14.4%) 351 BCE 311 BCE (81.1%) 202 BCE
	UBA-35774	GU-6	2178 ± 26	331 BCE (3.5%) 286 BCE 257 BCE (1.1%) 244 BCE 236 BCE (90.8%) 96 BCE
	UBA-41851	GE-C2	1982 ± 28	50 BCE (94.8%) 82 CE 100 CE (0.7%) 106 CE
	UBA-41852	GE-C3	1793 ± 30	104 CE (95.4%) 239 CE
	UBA-35775	GU-7	1866 ± 25	142 CE (95.4%) 244 CE

	UBA-41853	GE-C4	1693 ± 30	250 CE (52.8%) 296 CE 316 CE (42.7%) 398 CE
	UBA-41854	GE-C5	1674 ± 30	336 CE (77.9%) 436 CE 462 CE (2.4%) 478 CE 494 CE (15.1%) 537 CE
Tremml-Schacht-413				
	UBA-41725	TREM-2	199 ± 24	1638 CE (95.4%) 1800 CE
	UBA-41724	TREM-1	149 ± 21	1646 CE (95.4%) 1808 CE
Großer Naturschacht				
	UBA-41466	GNS-C4	239 ± 21	1638 CE (58.9%) 1676 CE 1742 CE (1.3%) 1750 CE 1766 CE (35.3%) 1800 CE
	UBA-41468	GNS-C6	155 ± 24	1659 CE (55.0%) 1708 CE 1725 CE (40.4%) 1858 CE
	UBA-41470	GNS-C8	191 ± 20	1646 CE (70.9%) 1690 CE 1761 CE (24.5%) 1808 CE
	UBA-38835	K6	107 ± 24	1687 CE (70.5%) 1744 CE 1800 CE (25.0%) 1836 CE
	UBA-41472	GNS-C10	166 ± 20	1796 CE (53.9%) 1826 CE 1836 CE (41.6%) 1902 CE
	UBA-41473	GNS-C11	65 ± 23	1806 CE (54.2%) 1834 CE 1845 CE (41.3%) 1909 CE
	UBA-41474	GNS-C12	83 ± 22	1810 CE (54.0%) 1840 CE 1848 CE (41.4%) 1915 CE
	UBA-41469	GNS-C7*	155 ± 24	1666 CE (16.1%) 1704 CE 1719 CE (31.1%) 1784 CE 1794 CE (9.8%) 1818 CE 1832 CE (18.5%) 1891 CE 1906 CE (19.9%) 1948 CE
	UBA-41471	GNS-C9*	20 ± 19	1700 CE (26.6%) 1723 CE 1814 CE (26.3%) 1834 CE 1888 CE (42.6%) 1910 CE
Eisgruben				
	UBA-39212	EG-1	4703 ± 29	3621 BCE (5.2%) 3582 BCE 3533 BCE (20.3%) 3484 BCE 3474 BCE (70.0%) 3370 BCE
	UBA-41969	EE-C1	4105 ± 40	2873BC (27.5%) 2796BC 2782BC (66.1%) 2570BC 2518BC (1.9%) 2501BC
	UBA-41970	EE-C2	1750 ± 28	235 CE (95.4%) 401 CE
	UBA-41971-	EE-C3	1072 ± 25	893 CE (21.3%) 928 CE 945 CE (74.1%) 1025 CE
	UBA-41972	EE-C4*	51 ± 27	1694 CE (24.7%) 1725 CE 1810 CE (27.5%) 1860 CE 1876 CE (30.8%) 1916 CE 1946 CE (12.4%) 1962 CE
	UBA-41973	EE-C5	2148 ± 27	156 BCE (95.4%) 80 CE
	UBA-41975	EE-C7	1646 ± 28	265 CE (0.7%) 272 CE 334 CE (93.4%) 443 CE 461 CE (1.4%) 475 CE
	UBA-41985	EE-C17	1492 ± 30	548 CE (95.4%) 640 CE
	UBA-41987	EE-C19	1203 ± 22	779 CE (95.4%) 880 CE

	UBA-41988	EE-C20	1028 ± 24	985 CE (94.7%) 1044 CE 1085 CE (0.7%) 1092 CE
	UBA-39213	EG-3	2019 ± 23	102 BCE (95.4%) 64 CE
	UBA-39214	EG-4	1894 ± 24	81 CE (5.2%) 100 CE 107 CE (90.2%) 217 CE
	UBA-41976	EE-C8	1196 ± 26	774 CE (84.9%) 896 CE 921 CE (10.5%) 956 CE
	UBA-41977	EE-C9	1025 ± 30	986 CE (54.7%) 1048 CE 1080 CE (40.8%) 1154 CE
	UBA-41979	EE-C11	776 ± 21	1226 CE (95.4%) 1277 CE
	UBA-41980	EE-C12	704 ± 24	1276 CE (95.4%) 1304 CE
	UBA-41981	EE-C13	650 ± 23	1276 CE (95.4%) 1304 CE
	UBA-41982	EE-C14	592 ± 27	1344 CE (8.6%) 1369 CE 1376 CE (86.9%) 1422 CE
	UBA-41983	EE-C15	324 ± 23	1480 CE (95.4%) 1584 CE
	UBA-41984	EE-C16	339 ± 21	1518 CE (1.8%) 1527 CE 1538 CE (93.6%) 1640 CE
	UBA-41978	EE-C10*	1124 ± 26	777 CE (0.7%) 782 CE 878 CE (94.7%) 994 CE
	UBA-41986	EE-C18*	1127 ± 27	775 CE (1.5%) 784 CE 834 CE (1.4%) 845 CE 876 CE (92.6%) 994 CE
	UBA-41974	EE-C6*	2067 ± 25	164 BCE (84.6%) 29 BCE 20 BCE (10.8%) 8 CE
Hochschneid				
	UBA-43647	HE-C01	541 ± 26	1390 CE (95.4%) 1427 CE
	UBA-35176	K	509 ± 27	1416 CE (95.4%) 1446 CE
	UBA-43986	HE-C03	487 ± 27	1416 CE (95.4%) 1446 CE
	UBA-43987	HE-C04	426 ± 40	1430 CE (95.4%) 1474 CE
	UBA-35177	L	366 ± 31	1460 CE (95.4%) 1524 CE
	UBA-35178	M	387 ± 28	1495 CE (11.0%) 1530 CE 1536 CE (84.5%) 1608 CE
	UBA-43990	HE-C08	298 ± 21	1520 CE (94.3%) 1604 CE 1616 CE (1.1%) 1624 CE
	UBA-43991	HE-C10	364 ± 19	1582 CE (95.4%) 1635 CE
	UBA-43641	HE-C11	257 ± 28	1606 CE (95.4%) 1660 CE
	UBA-43992	HE-C12	215 ± 21	1642 CE (94.3%) 1694 CE 1740 CE (0.9%) 1748 CE 1784 CE (0.3%) 1787 CE
	UBA-43993	HE-C13	158 ± 22	1666 CE (94.4%) 1768 CE 1800 CE (1.0%) 1808 CE
	UBA-43988	HE-C05*	271 ± 20	1522 CE (32.7%) 1574 CE 1626 CE (60.7%) 1664 CE 1785 CE (2.1%) 1794 CE
	UBA-43989	HE-C06*	460 ± 19	1424 CE (95.4%) 1455 CE
	UBA-43639	HE-C07*	281 ± 27	1507 CE (55.1%) 1594 CE 1616 CE (39.1%) 1664 CE 1786 CE (1.3%) 1794 CE
	UBA-43640	HE-C09*	265 ± 25	1520 CE (29.6%) 1579 CE 1624 CE (58.6%) 1669 CE 1780 CE (7.3%) 1798 CE

Kraterschacht				
	UBA-38830	K1	650 ± 34	1280 CE (38.5%) 1330 CE<br>1335 CE (57.0%) 1398 CE
	UBA-38831	K2	336 ± 30	1472 CE (95.4%) 1598 CE
	UBA-38833	K4	120 ± 28	1680 CE (21.2%) 1740 CE<br>1752 CE (1.6%) 1763 CE<br>1800 CE (72.7%) 1944 CE
	LTL4510A	P3*	886 ± 45	1036 CE (93.5%) 1230 CE<br>1244 CE (1.9%) 1256 CE
